# Quantitative systems pharmacology modeling of class I–III BRAF-mutant NSCLC enables subgroup treatment analysis and combination therapy design

**DOI:** 10.1016/j.isci.2026.117854

**Published:** 2026-10-13

**Authors:** Jingjing Hu, Qi Rao, Zichun Li, Geli Li, Zhenle Bi, Danyi Wen, Chen Zhao, Guohua Zhou

**Affiliations:** 1 Department of Clinical Pharmacy, Jinling Hospital, Affiliated Hospital of Medical School, Nanjing University, Nanjing, China; 2 School of Pharmacy, Nanjing Medical University, Nanjing, China; 3 Clinical Trial Centre, Sichuan Academy of Medical Sciences & Sichuan Provincial People’s Hospital, Chengdu, Sichuan, China; 4 School of Chemical, Materials and Biomedical Engineering, University of Georgia, Athens, Georgia, USA; 5 Shanghai LIDE Biotech Co., Ltd., Shanghai, China; 6 Department of Oncology, The First Affiliated Hospital with Nanjing Medical University, Nanjing, China

**Keywords:** BRAF, quantitative systems pharmacology, rare mutations, virtual clinical trials, combination therapy, drug resistance

## Abstract

BRAF-mutant non-small cell lung cancer (NSCLC) comprises three functional classes (I–III) with distinct driving mechanisms, yet effective therapies for classes II and III remain limited. To address this, we developed a quantitative systems pharmacology (QSP) model that mechanistically recapitulates signaling networks and drug responses across all three classes, integrating multiscale preclinical-to-clinical datasets. The model enabled high-throughput prediction of clinical efficacy for monotherapies and combination therapies across diverse dosing regimens, and we identified that combining MEK and ERK inhibition represents a novel strategy for class II/III mutants, validated by original *in vivo* MiniPDX data using NSCLC patient tumors. Simulations further revealed that only triple blockade of EGFR, IGF1R, and FGFR pathway initiation can potently reduce tumor growth and overcome resistance in these classes. Our work is the first multiscale QSP platform mechanistically addressing class I–III BRAF mutations at the systems level for disease-centric translational combination therapy discovery and precision medicine design.

## Highlights

• A QSP modeling platform integrating pathophysiology of class I–III BRAF-mutant NSCLC
• Model incorporated multiscale datasets of pathway signaling and intervention efficacy
• Model-predicted new combination therapies were validated by MiniPDX experiments
• Model simulations guide translational drug research and subgroup-based precision treatment

## Graphical abstract

**Figure:**
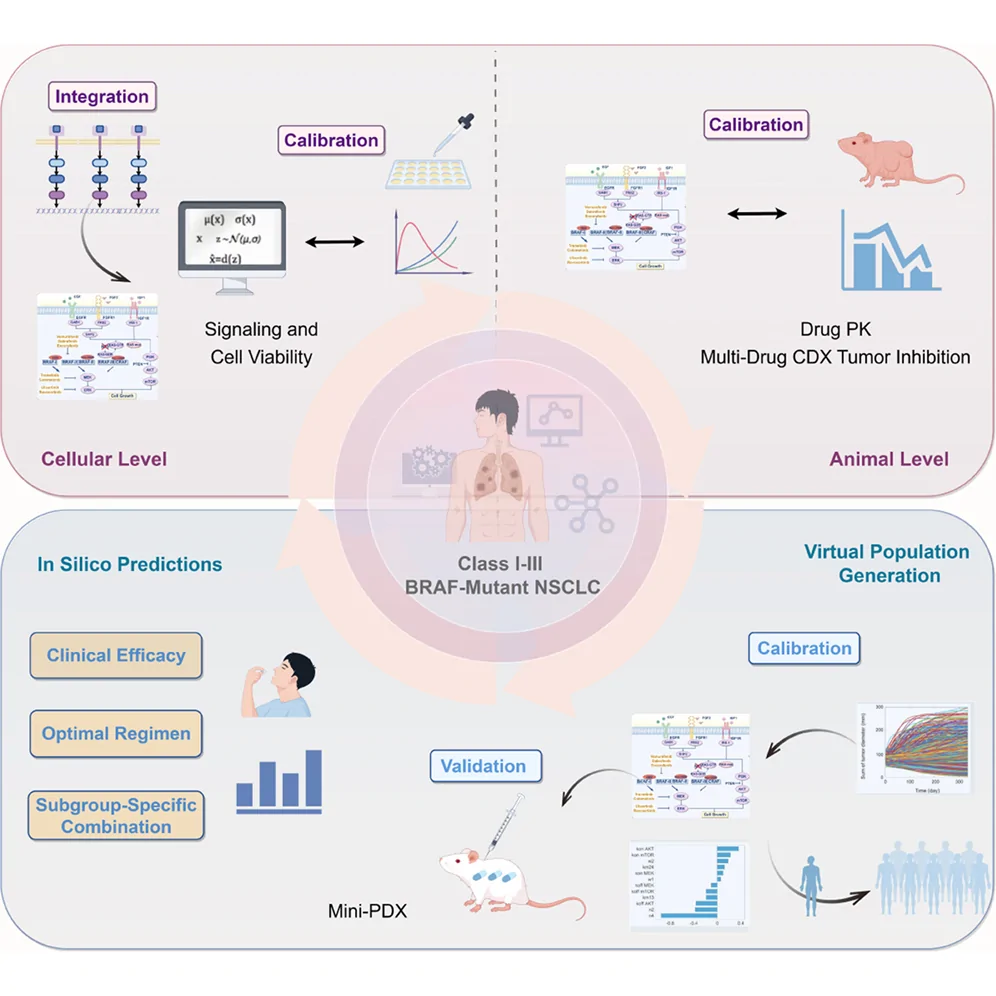

## Teaser

Oncology; Pharmacology

## Introduction

BRAF is a member of the rapidly accelerated fibrosarcoma (RAF) family of serine/threonine kinases and plays a critical role in cell proliferation and differentiation through the mitogen-activated protein kinase (MAPK) signaling pathway.^1^^,^^2^ Mutated BRAF can function as an oncogene, thereby contributing to tumorigenesis.^3^ Approximately 2.2%–4.9%^4^^,^^5^ of patients with advanced non-small cell lung cancer (NSCLC) have BRAF mutations. The considerable heterogeneity of mutation subtypes influences the clinical and biological characteristics of BRAF-mutant NSCLC. Recent studies have classified BRAF mutations into three categories according to their genomic location and functional activity.^6^ Class I BRAF mutants consist of single nucleotide variations at codon 600, primarily p.V600E, and function as constitutively active kinases that signal independently of their dimerization status. Class II BRAF mutations are characterized by constitutive dimerization, which confers RAS-independent signaling and results in moderate to robust kinase activity.^7^ In contrast, class III BRAF mutations, which have low or absent kinase activity, can drive tumorigenesis through enhanced binding to RAS and by forming heterodimers with wild-type RAF isoforms.^8^ Class III BRAF mutations frequently coexist with RAS mutations or loss-of-function alterations in NF1.^6^ BRAF mutations are categorized in clinical practice as either V600 or non-V600 mutations. Non-V600 mutations include all class II and class III variants and account for approximately 50% of BRAF mutations in NSCLC.^9^^,^^10^^,^^11^ As a result, drug development initiatives in this field are actively ongoing, focusing on innovative combination strategies and novel targeted therapies.

Small molecule inhibitors are the main therapeutic modality for BRAF-mutant cancers.^12^ MEK inhibitors (MEKi) (such as trametinib and cobimetinib), RAF inhibitors (RAFi) (such as vemurafenib, dabrafenib, and encorafenib), and ERK inhibitors (ERKi) (such as ulixertinib and ravoxertinib) have been developed and implemented in clinical practice, mostly for class I BRAF-mutant cancers. These molecules have demonstrated meaningful clinical efficacy and received approval for use in various BRAF-mutant malignancies, including advanced melanoma,^13^ recurrent or metastatic thyroid cancer,^14^ and colorectal cancer.^15^^,^^16^ However, in advanced NSCLC harboring BRAF mutations, research remains relatively limited, and treatment options are constrained. Currently, the combination of dabrafenib and trametinib is approved only as a first-line regimen for patients with advanced NSCLC carrying class I BRAF V600E mutations.^17^^,^^18^ In contrast, no effective targeted therapies are available for patients with class II or class III BRAF mutations, resulting in considerable uncertainty and limited options regarding treatment strategies for these patients. This therapeutic impasse primarily stems from the distinct and complex biological characteristics of class II and III BRAF mutations. Unlike class I mutations, class II and III BRAF mutations typically do not respond to conventional BRAF inhibitor monotherapy or combination regimens and frequently drive tumor proliferation and survival through multiple compensatory signaling pathways. Specifically, adaptive resistance mechanisms include loss of NF1 function, sustained reactivation of the RAS-MAPK pathway due to acquired RAS mutations,^6^ and bypass signaling via receptor tyrosine kinases (RTKs) such as FGFR or IGF1R,^19^ which can all contribute to treatment failure. These mechanisms collectively sustain baseline MAPK pathway output and may synergistically activate the PI3K-AKT pathway,^20^ thereby undermining the efficacy of existing targeted therapies. Unfortunately, clinical data on class II and III BRAF-mutant NSCLC are substantially scarcer than those for class I mutations, greatly impeding the advancement of translational research and the development of targeted strategies. Consequently, there is a pressing need to develop therapies that target non-V600E BRAF mutations and their compensatory networks.

Considering the intricate mechanisms of BRAF mutations in the progression of NSCLC across class I, II, and III mutations, along with the consistently elevated costs of novel therapies during clinical trials, quantitative systems pharmacology (QSP) modeling—representing a contemporary paradigm in model-based drug development—arises as a potent methodology to drive drug development in this field. Through the integration of multi-level mechanisms and multi-modal data, QSP modeling can facilitate quantitative predictions of therapeutic efficacy, therefore expediting translational drug development and the identification of innovative clinical treatment regimens.^21^^,^^22^ In the field of NSCLC drug research, QSP modeling has been increasingly investigated during the drug development phase to facilitate clinical trial design, particularly for immuno-oncology drug candidates.^23^^,^^24^ Nonetheless, in the investigation of BRAF mutation-targeted therapies, only Kirouac et al.^25^ formulated a systems biology model for BRAF V600E-mutant colorectal cancer. Thus far, no QSP modeling attempts have been conducted with the goal of enhancing precision medicine or discovering new treatment plans for class II and III BRAF mutations. Thus, we have developed a QSP model for NSCLC that contains all three classes of BRAF mutations. This model includes intricate mechanistic features that extend from the clinical patient level to complex cellular signaling pathways. The QSP model underwent thorough calibration and validation using comprehensive experimental datasets, which encompassed signal transduction, cell viability, pharmacokinetics, tumor growth inhibition, and clinical-level treatment responses for more than 7 candidate drugs. Utilizing this model, we examined the prospective therapeutic impacts of various treatment protocols and combination strategies among different patient subgroups of class I–III mutations. We found that the combination of MEKi and ERKi can greatly improve therapeutic response in class II and III BRAF mutations, and this model prediction was validated by original efficacy data using the *in vivo* MiniPDX technology with tumor tissues from actual class II and III BRAF-mutant NSCLC patients. Furthermore, the model provided novel insights into overcoming RTK-driven resistance, demonstrating that a triple combination targeting EGFR, IGF1R, and FGFR1 was necessary to achieve substantial tumor regression in both class II and III mutant settings, surpassing the limited efficacy of dual EGFR/IGFR blockade. This study constitutes the first multiscale QSP modeling initiative for class I–III BRAF mutations, and it creates a translational data-driven model-informed platform for future anti-cancer drug research.

## Results

### Overview of QSP model structure and mechanisms

The comprehensive QSP model comprises a cell signaling module and several pharmacokinetic modules, designed to thoroughly elucidate how class I, class II, and class III BRAF mutations facilitate tumor proliferation and how targeted pathway interventions modulate tumor growth (Figure 1). The distinct signaling mechanisms of the three BRAF mutation classes were incorporated into the model based on current pathophysiological understanding. Class I mutations are defined as RAS-independent monomers with high kinase activity, whereas class II mutations function as RAS-independent dimers with moderate activity. Class III mutants, in contrast, are kinase-impaired, and they signal primarily through mutant BRAF-wild-type CRAF heterodimers. For all three classes, pathway activation is subject to negative feedback inhibition mediated by ERK, which acts on GTP-bound RAS. The activation intensity of downstream pathways for all classes was calibrated using experimental data from Rajkumar et al.^26^ The cellular signaling module delineates the pro-tumorigenic intracellular pathways and interactions co-regulated by the RTKs EGFR, FGFR1, and IGF1R. Specifically, the binding of EGF, FGF2, and IGF1 to EGFR, FGFR1, and IGF1R results in significant phosphorylation of these receptors, which in turn activates the downstream PI3K/AKT and RAF/MAPK signaling pathways, thereby facilitating cancer cell proliferation. The model includes the FGFR1 ligand-independent self-activation mechanism, in which FGFR1 can initiate downstream signal transduction cascades via self-phosphorylation without the presence of FGF2.^27^ In addition, the model also examines the impact of the coexistence of class III BRAF mutations with RAS mutations or NF1 loss on downstream signaling pathways associated with cell proliferation. We integrated the mechanisms of action of various candidate drugs from three distinct classes (MEKi, RAFi, and ERKi) into the QSP model, including trametinib, cobimetinib, vemurafenib, dabrafenib, encorafenib, ulixertinib, and ravoxertinib. The differential pharmacokinetic processes of the drugs are characterized by compartmental models, and these agents are presumed to directly interact with their respective targets to downregulate protein phosphorylation. Information regarding the model species, reactions, and descriptions is available in Table S1.

**Figure 1:**
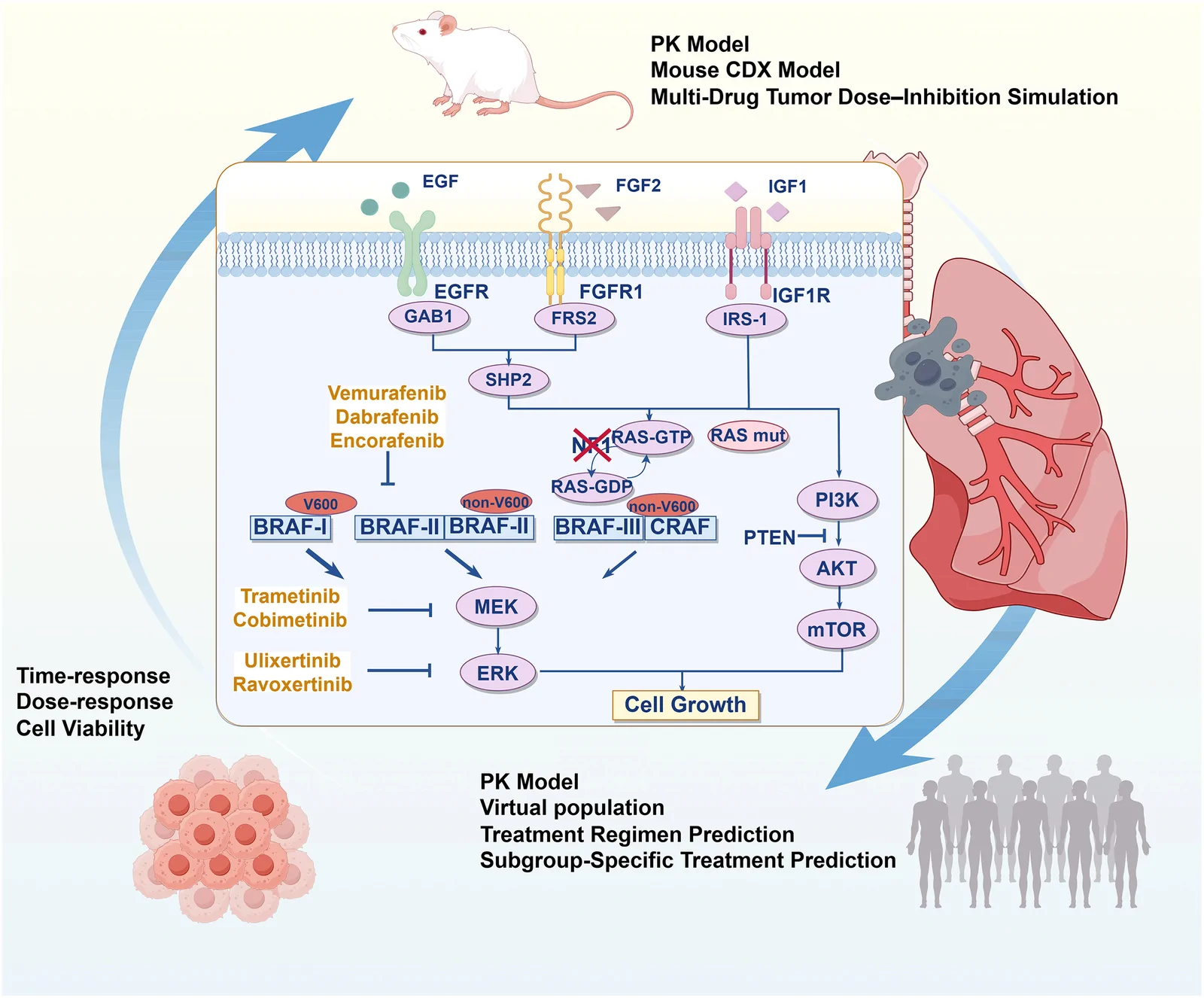
Overview of the QSP model and the process of multiscale model calibration, validation, and analysis A mechanism-based multiscale QSP model was created to evaluate intervention regimens for NSCLC patients with class I, II, and III BRAF mutations. Key receptor tyrosine kinase pathways associated with NSCLC cell proliferation, including EGFR, FGFR1, and IGF1R, were integrated and validated using multi-modal *in vitro* data. The cell-level model was subsequently expanded to the preclinical *in vivo* stage by incorporating pharmacokinetic elements of various drugs, and the model was calibrated to tumor growth profiles across multiple treatment scenarios in mice. Then at the clinical level, the QSP model was revised and employed to generate virtual patient populations capable of accurately simulating clinical efficacy outcomes for various targeted therapies, as well as to assess novel combination treatment strategies and dose optimization possibilities.

### Stepwise model calibration and validation utilizing multimodal cellular signaling and drug intervention data under class I–III BRAF mutant scenarios

The cell signaling module in this QSP model was predominantly calibrated/validated using data obtained from NSCLC cell lines with class I (HCC364 cells), class II (NCI-H1395 and NCI-H1755 cells), and class III BRAF mutations (NCI-H1666 and CAL-12T cells). Before any ligand stimulation or drug treatment, all protein levels in the cell signaling module were set to their equilibrium states. Subsequently, various experimental interventions were incorporated into the model, and the simulated outputs were directly compared with the corresponding *in vitro* data obtained under the same experimental conditions. To this end, a large amount of multimodal experimental data was analyzed, which encompassed ligand-dependent activation (e.g., EGF, FGF2, and IGF1), drug-induced inhibition of intracellular signaling, and drug-induced regulation of cancer cell survival (including time-dependent and dose-dependent characteristics involving seven drugs). Specifically, the analysis included around 121 sets (with over 605 data points) of *in vitro* experimental data, involving 7 drugs, such as MEKi (e.g., trametinib and cobimetinib), RAFi e.g., vemurafenib, dabrafenib, and encorafenib), and ERKi (e.g., ulixertinib and ravoxertinib).

The QSP model integrates class I, II, and III BRAF mutation mechanisms, quantitatively replicating data on total EGFR^28^ (Figure 2A), EGFR phosphorylation^28^ (Figures 2B and 2C), GAB1 phosphorylation^28^ (Figures 2D and 2E), and SHP2 phosphorylation levels^29^ (Figure 2F) across various EGF stimulation conditions. These results correspond with quantitative experimental observations in class III mutant cells. Further optimization was also conducted utilizing experimental data from EGF-mediated activation of the EGFR pathway in additional cancer cell lines, encompassing alterations in total EGFR protein levels (Figures S1A–S1D), EGFR phosphorylation levels (Figure S1E), and downstream proteins including SHP2 (Figure S1F), RAS-GTP (Figures S1G and S1H), RAF (Figure S1I), MEK (Figures S1J–S1M), ERK (Figure S1N), AKT^30^^,^^31^ (Figures 2G and 2H) and mTOR (Figures S1O and S1P). Subsequently, model optimization was done for the IGF pathway, including IGF1-mediated alterations in total IGF1R protein levels^32^ (Figure 2I; Figures S1Q–S1T), IGF1R phosphorylation levels^32^ (Figures 2J, S1U, and S1Vi), and downstream activation of IRS-1 (Figure S1Vii), MEK (Figure S1Viii), ERK^32^ (Figures 2K and S2W), PI3K (Figure S2Wi), and AKT protein^32^ (Figures 2L, 2M, S2Wii, and S2Xi). Finally, model optimization was done for the FGF pathway, including FGF2-mediated alterations in total FGFR1 protein levels^33^ (Figures 2N, S2Xii, and S2Xiii), FGFR1 phosphorylation levels^33^^,^^34^ (Figures 2O–2Q), and downstream MEK (Figure S2Y), ERK^35^ (Figures 2R–2T), and AKT proteins (Figure S2Z).

**Figure 2:**
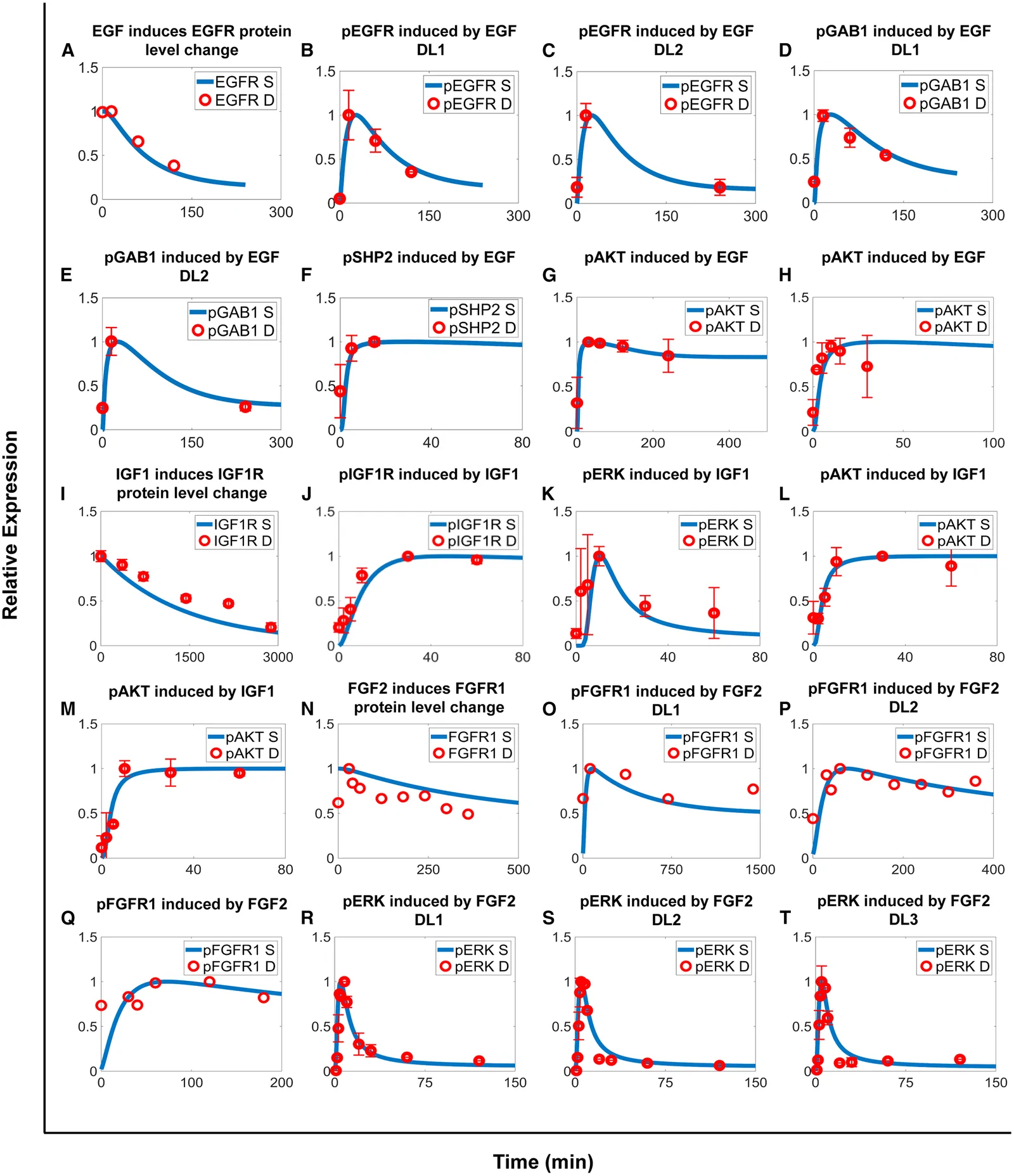
Calibration of the cell-level QSP model using comprehensive pathway signal transduction data from BRAF-mutant cancer cells In the class III BRAF-mutant NSCLC cell line, (A) EGF (10 ng/mL) induces dimerization, internalization, and degradation of EGFR, along with (B and C) phosphorylation-dependent activation of EGFR (B: 10 ng/mL, C: 38 ng/mL). (D and E) EGF (D: 10 ng/mL, E: 38 ng/mL) induces downstream activation of GAB1. (F) EGF (10 ng/mL) induces downstream activation of SHP2. In cancer cells with normal BRAF, (G and H) EGF (G: 100 ng/mL, H: 50 ng/mL) induces downstream activation of AKT. For the IGF1R pathway, (I) IGF1 (50 ng/mL) induces dimerization, internalization, and degradation of IGF1R, along with (J) phosphorylation and activation of IGF1R, (K) ERK activation, and (L and M) AKT activation. For the FGFR1 pathway, (N) FGF2 (25 ng/mL) induces dimerization, internalization, and degradation of FGFR1. (O–Q) FGF2 induces downstream activation of FGFR1 (O: 20 ng/mL, P, Q: 25 ng/mL). (R–T) FGF2 induces downstream activation of ERK (R: 20 ng/mL, S: 100 ng/mL, T: 500 ng/mL). (A–T) *y* axes are relative expression levels (normalized to their respective maximum values). S, simulation; D, experimental *in vitro* data; DL, dose level.

Next, the QSP model was optimized by calibrating the inhibitory effects of various targeted inhibitors at different concentrations on intracellular signaling and cell survival in BRAF-mutant NSCLC cell lines. In the BRAF class I mutation scenario, the model quantitatively replicated the downregulation of MEK and ERK phosphorylation levels, as well as the inhibition of cell viability observed in the class I BRAF mutant cell line (HCC364) after administration of the MEKi trametinib and cobimetinib^19^^,^^36^ (Figures 3A–3D, S3A, and S3B). The model was further calibrated utilizing data on the dependent inhibitory effects of RAFi vemurafenib, dabrafenib, and encorafenib on MEK and ERK activation, as well as their collective influence on cell viability^19^^,^^36^^,^^37^ (Figures 3E–3I, S3C, and S3D). We also calibrated the model using experimental data that demonstrated the dose-response inhibitory effects of the ERKi ulixertinib and ravoxertinib on cell viability (Figures S3E and S3F). To assess the predictive performance under the class I mutation scenario, we simulated the impact of vemurafenib in conjunction with cobimetinib on cellular viability. The model predictions precisely captured the inhibition of cellular viability by the aforementioned drug combination^36^ (Figure 3J). In the class II BRAF mutation scenario, the dose-response curves for the RAFi vemurafenib, dabrafenib, and encorafenib were quantitatively recapitulated across several class II-mutant NSCLC cell lines (NCI-H1395 and NCI-H1755): as drug dose increases, MEK and ERK phosphorylation levels and cell viability would all decrease^36^^,^^37^ (Figures 3K–3N, 4A, 4B, and S3H–S3M). Additionally, the model accurately described the modulation of MEK and ERK by MEKi (trametinib and cobimetinib) in the NCI-H1395 and NCI-H1755 cell lines, as well as the suppression of cell viability^36^ (Figures 4C, 4D, and S3N–S3P). Furthermore, we calibrated the model using data on the suppression of cellular viability by ERKis (ulixertinib and ravoxertinib)^36^ (Figures 4E, 4F, S3Q, and S3R). To assess the predictive capacity under the class II mutation scenario, we simulated the synergistic effects of vemurafenib and encorafenib on the regulation of MEK and ERK^38^ (Figures 4G and 4H), and the model predictions were in good agreement with experimental results. We also validated the model using combination therapy data, including vemurafenib with cobimetinib and trametinib with dabrafenib. The model simultaneously replicated the intricate nonlinear inhibitory effects of various drug combinations on cell survival^36^ (Figures 4I and S3S–S3U). In the class III mutation scenario, we utilized data on the regulation of ERK by RAFi dabrafenib and vemurafenib in several class III mutant NSCLC cell lines (NCI-H1666 and CAL-12T) and their inhibitory effects on cell viability to calibrate the model^19^^,^^37^^,^^39^ (Figures 4J–4P and S4V). The model quantitatively captured the downregulation of ERK and the suppression of cell survival by trametinib^19^ (Figures 5A–5C). We again validated the model in this scenario using data from combination therapies of trametinib plus dabrafenib and vemurafenib plus encorafenib. The model accurately predicted the inhibitory effects of various drug combinations on cell viability as obtained from *in vitro* experiments^40^ (Figures 5D and S4Vi). Given that patients with class III mutations may also possess RAS mutations or NF1 deletions, we also integrated mechanisms for the co-occurrence of class III mutations with RAS mutations or NF1 deletions into our model. Calibration of this module has utilized data from the class III BRAF mutation and RAS mutation cell line SK-MEL-208, and both the model and data indicated downregulation of MEK and ERK after treatment with vemurafenib (Figures S4Viii and S4W). The model was also calibrated using data on the regulation of ERK by trametinib and encorafenib, demonstrating high predictive accuracy against experimental data (Figures S4Vii and S4Wi). Moreover, the QSP model was calibrated using data on ERK regulation after encorafenib treatment in various class III BRAF mutation cell lines with NF1 deletion (NZM041 and WM3912) (Figures S4Wii and S4Wiii). The overall results demonstrated the significant potential of the cellular signaling modules within our QSP model for predicting dynamic changes in cellular signaling and the effects of drug inhibition under complex targeted interventions for various class I–III BRAF mutation scenarios.

**Figure 3:**
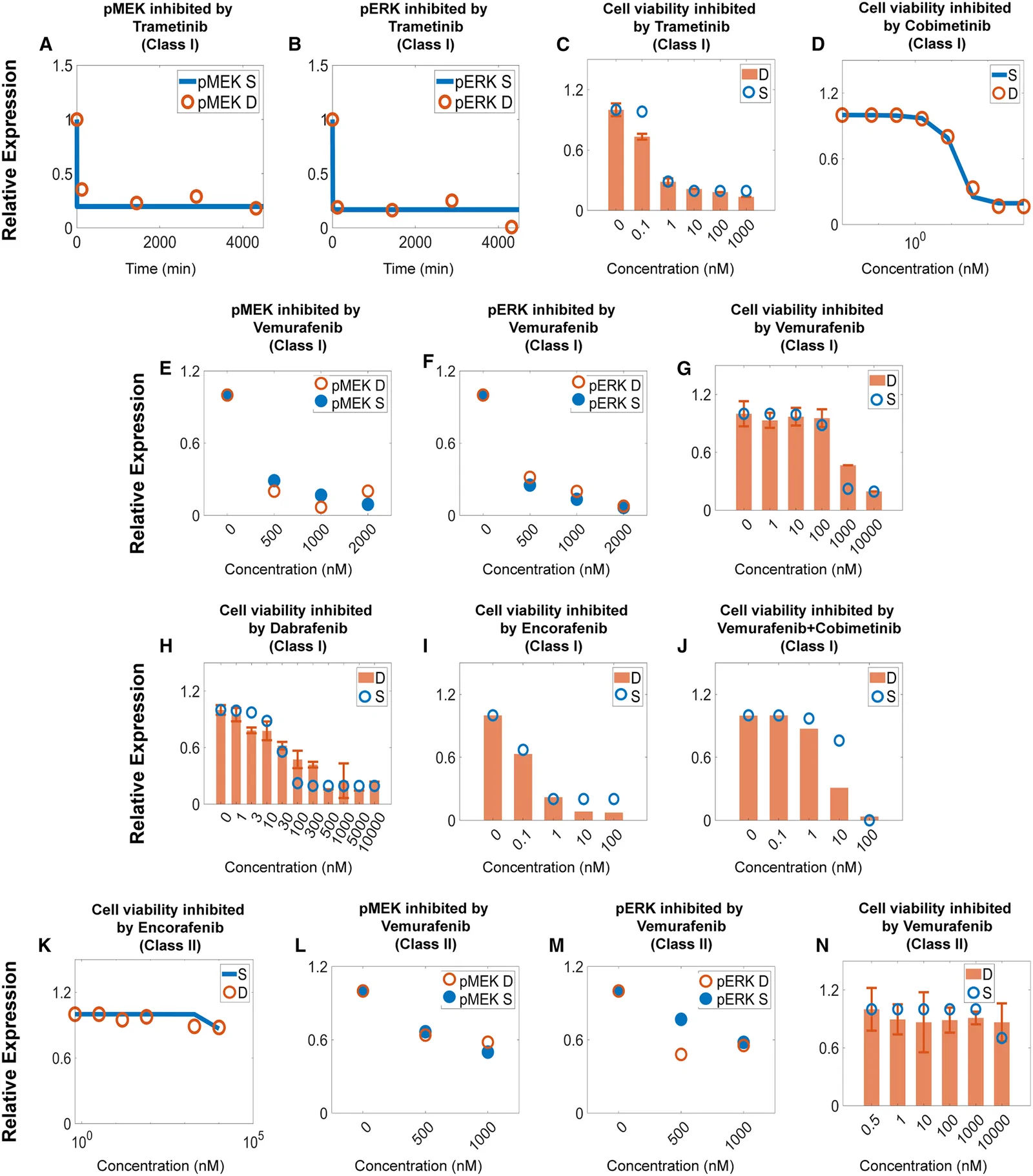
Cell-level QSP model calibration and validation using drug intervention and cell viability data from class I/II BRAF mutation cell lines In the class I BRAF-mutant cell line, trametinib (MEKi) induces time- and dose-dependent inhibition of (A) MEK and (B) ERK activation, as well as (C) cell viability. Cobimetinib (MEKi) induces dose-dependent inhibition of (D) cell viability. Vemurafenib (RAFi) inhibits (E) MEK and (F) ERK activation, as well as (G) cell viability in a dose-dependent manner. (H) Dabrafenib (RAFi), (I) encorafenib (RAFi), and (J) the vemurafenib (RAFi) plus cobimetinib (MEKi) combination all inhibit cell viability in a dose-dependent manner. In the class II BRAF-mutant cells, encorafenib (RAFi) induces dose-dependent inhibition of (K) cell viability. Vemurafenib (RAFi) inhibits (L) MEK and (M) ERK activation and (N) cell viability in a dose-dependent manner. (A–N) *y* axes are relative expression levels (normalized to their respective maximum values or the no-treatment condition). S, simulation; D, experimental *in vitro* data.

**Figure 4:**
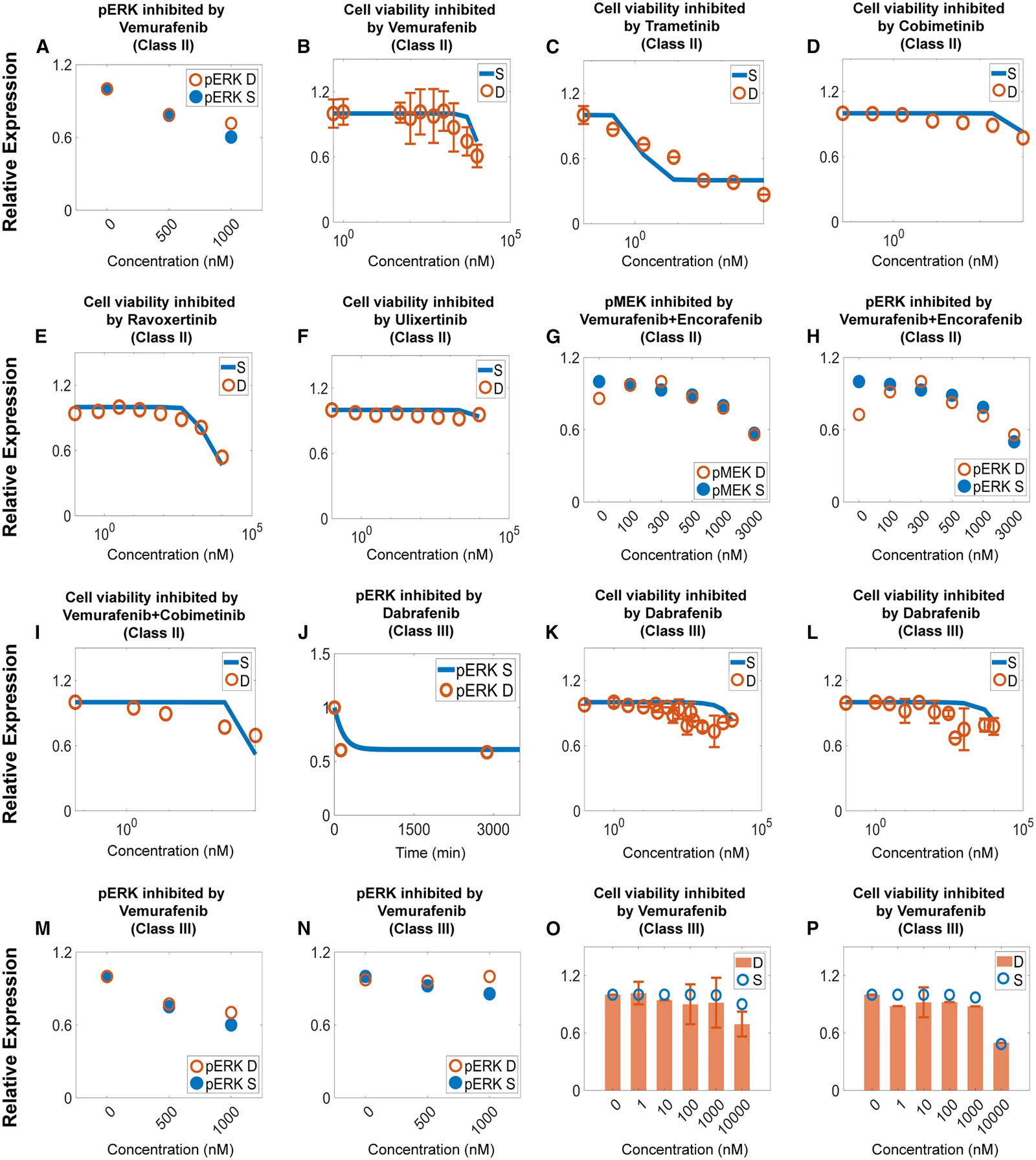
Additional calibration and validation of the cell-level QSP model using drug intervention and cell viability data from class II/III BRAF mutation cell lines In the class II BRAF-mutant cells, vemurafenib (RAFi) induces dose-dependent inhibition of (A) ERK activation and (B) cell viability. (C) Trametinib (MEKi), (D) cobimetinib (MEKi), (E) ravoxertinib (ERKi), and (F) ulixertinib (ERKi) inhibit cell viability in a dose-dependent manner. The vemurafenib (RAFi) plus encorafenib (RAFi) combination induces dose-dependent inhibition of (G) MEK and (H) ERK activation. The vemurafenib (RAFi) plus cobimetinib (MEKi) combination inhibits (I) cell viability in a dose-dependent manner. In the class III BRAF-mutant cells, dabrafenib (RAFi) induces time- and dose-dependent inhibition of (J) ERK activation and (K and L) cell viability. Vemurafenib (RAFi) inhibits (M and N) ERK activation and (O and P) cell viability in a dose-dependent manner. (A–P) *y* axes are relative expression levels (normalized to their respective maximum values or the no-treatment condition). S, simulation; D, experimental *in vitro* data.

**Figure 5:**
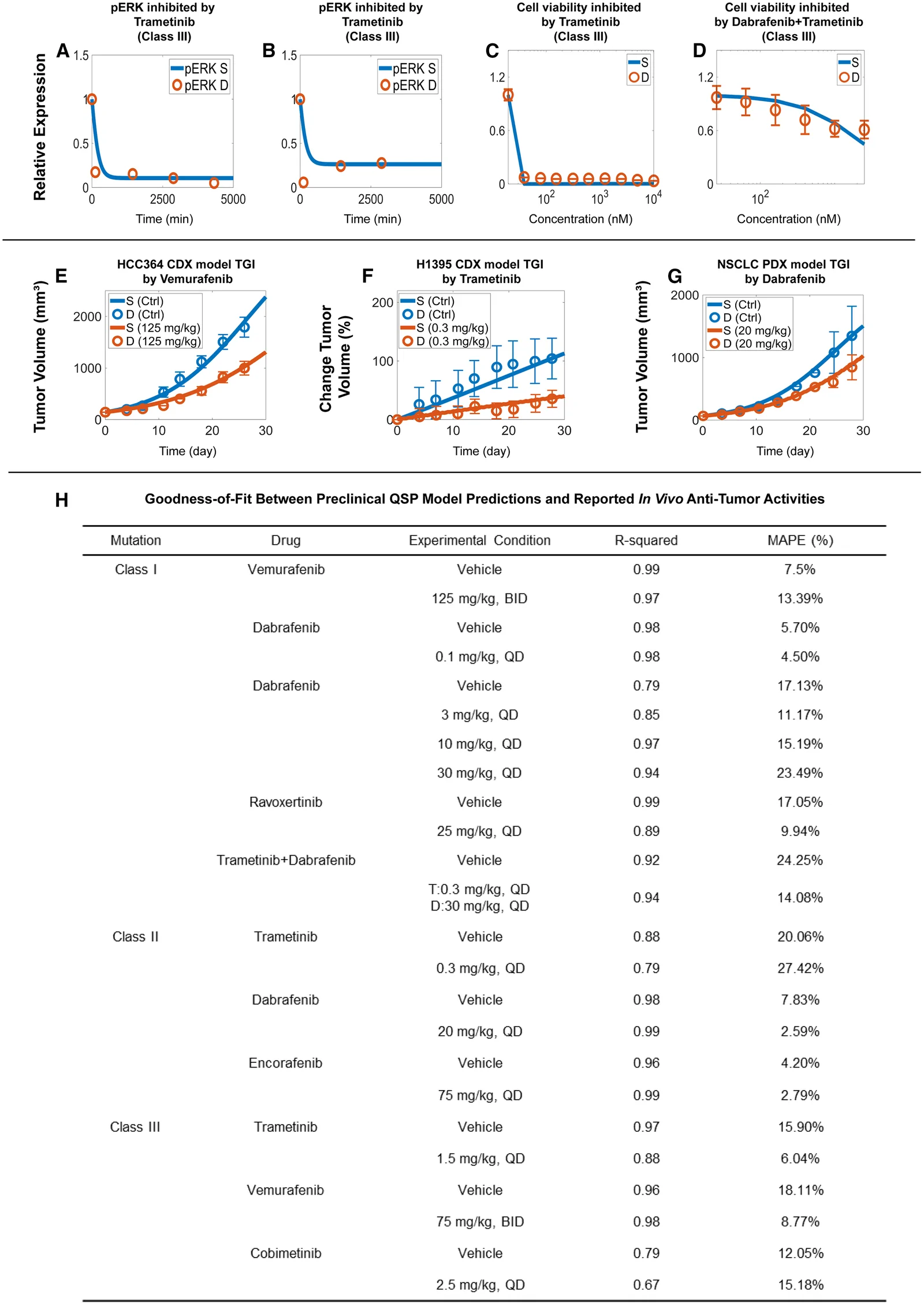
Translational QSP model calibration and validation using preclinical data on drug-induced cell viability and *in vivo* tumor growth inhibition In the class II BRAF-mutant cells, trametinib induces dose-dependent inhibition of (A and B) ERK activation and (C) cell viability. Dabrafenib (RAFi) plus trametinib (MEKi) combination inhibits (D) cell viability in a dose-dependent manner. QSP model accurately replicates reported *in vivo* antitumor activity of (E) vemurafenib (RAFi) and (F) trametinib (MEKi) in a class I/II BRAF-mutant NSCLC cell-derived xenograft, and (G) dabrafenib (RAFi) in a class III BRAF-mutant NSCLC patient-derived xenograft model. (H) Goodness-of-fit (evaluated using R^2^ and MAPE metrics) between model simulations and experimental data across all *in vivo* studies. In the simulations, tumors were permitted to reach certain volumes prior to drug administration, as indicated by several referenced studies, with a maximum tumor volume established at 2,000 mm^3^. All tumor growth profiles were standardized to their initial volumes. The weight of a mouse was assumed to be approximately 20 g to calculate the drug doses administered. S, simulation; D, experimental *in vivo* data.

### QSP model-based quantitative evaluation of drug-induced suppression of tumor growth *in vivo*

Following thorough calibration and validation of the QSP model at the cellular signaling level, we subsequently evaluated the model’s predictive capability in the *in vivo* context. In the transition from *in vitro* to *in vivo*, we assumed that parameters associated with intracellular signaling and drug inhibition remained constant, whereas parameters related to cell growth and death could vary due to the differing microenvironments of tumors *in vivo*. Corresponding pharmacokinetic modules were developed for all drugs included in the *in vivo* analysis against drug concentration profiles obtained in mice. Following the calibration of tumor growth parameters based on the tumor growth curves from the control group (untreated), the resulting QSP model can effectively characterize the data on the temporal dynamics of tumor growth inhibition induced by seven drugs across thirteen doses *in vivo*.

The QSP model accurately reproduced the dose- and time-dependent inhibitory effects of drugs on tumor growth in various CDX models for class I BRAF mutations. Specifically, the model effectively simulated the growth curves of xenograft tumors originating from NSCLC (HCC364), thyroid carcinoma (KHM-5M), colorectal carcinoma (COLO205), and melanoma (A-375) cell lines^41^ (Figures 5E, S5A, S5B, and S5D–S5G). Notably, without adjusting any model parameters, the model also captured the synergistic antitumor impact of trametinib and dabrafenib in the A-375 CDX model (Figure S5C). For class II BRAF mutations, the model effectively characterizes the antitumor effects of trametinib in CDX models implanted with the NCI-H1395 cell line^19^ (Figure 5F) and quantitatively captured tumor growth profiles in NSCLC and melanoma patient-derived xenograft (PDX) models under dabrafenib and encorafenib treatment^42^ (Figures 5G and S5H). For class III BRAF mutations, the model quantitatively reproduced the tumor growth inhibition of trametinib, vemurafenib, as well as cobimetinib in PDX models (Figures S5I, S5J, and S5K). We further assessed the model’s predictive power against *in vivo* tumor growth inhibition data by quantifying the goodness-of-fit through R^2^ and mean absolute percentage error (MAPE) metrics. The results indicated that the majority of R^2^ values fell between 0.8 and 1, while MAPE remained consistently below 20%, demonstrating a robust correlation between predicted and observed values that can effectively qualify our QSP model (Figure 5H). PK fitting of the various drugs involved against mouse data was shown in Figure S6.

### Utilizing QSP model-based virtual clinical trials to predict patient response to different treatment plans across class I–III BRAF mutation scenarios

After rigorous preclinical calibration and validation, the QSP model was investigated at the clinical level by creating model-based virtual patient populations and conducting virtual clinical trials under different targeted treatments. PK fitting of the various drugs involved against clinical PK data was shown together in Figures S7 and S8. 3 virtual patient populations were successfully established, each consisting of 5,000 model-based virtual patients, to represent NSCLC patients with class I, class II, and class III BRAF mutations, respectively (refer to the STAR Methods section for details on virtual patient generation).

In the virtual patient population with class I mutations, we calibrated the virtual population using published clinical response data for vemurafenib and the vemurafenib plus cobimetinib combination in NSCLC patients (with class I BRAF mutation). From the virtual clinical trial results, we see that the simulated population-level response for the two treatment regimens, vemurafenib and vemurafenib plus cobimetinib, can accurately reproduce the actual clinical trial results (NCT01524978 and ACTRN12620000861954) in terms of objective response rate (ORR) (Figures 6A and 6B).^43^^,^^44^ We then validated this virtual clinical trial approach through predicting the clinical response to another RAFi dabrafenib, by adjusting only drug-specific efficacy parameters (from *in vitro* fittings) and human pharmacokinetic parameters. The model-predicted clinical treatment response to dabrafenib can again match the actual clinical trial data (NCT01336634) obtained from class I BRAF-mutant NSCLC patients (Figure 6A).^45^ We performed similar virtual patient population formulation and clinical trial simulation procedures for NSCLC patients with class II and III BRAF mutations (Figures 6C–6E). For example, our virtual clinical trials demonstrated diverse patient responses to vemurafenib as observed in actual individuals with class II mutations in the AcSé vemurafenib trial (Figure 6D),^46^ as well as the population-level patient response to trametinib as observed in patients with class III mutations in the NCI-MATCH trial.^47^ We also assessed the potential clinical efficacy, using virtual clinical trials, of treatment responses to single-agent MEKi or RAFi as well as their combination for patients with class I, II, and III BRAF mutations. The overall simulation results are in good agreement with literature clinical findings (of small sample sizes) regarding treatment response (e.g., ORR, disease control rate [DCR]) to the aforementioned therapies, specifically for response in patients with class II mutations (Figures 6F and 6G).^48^

**Figure 6:**
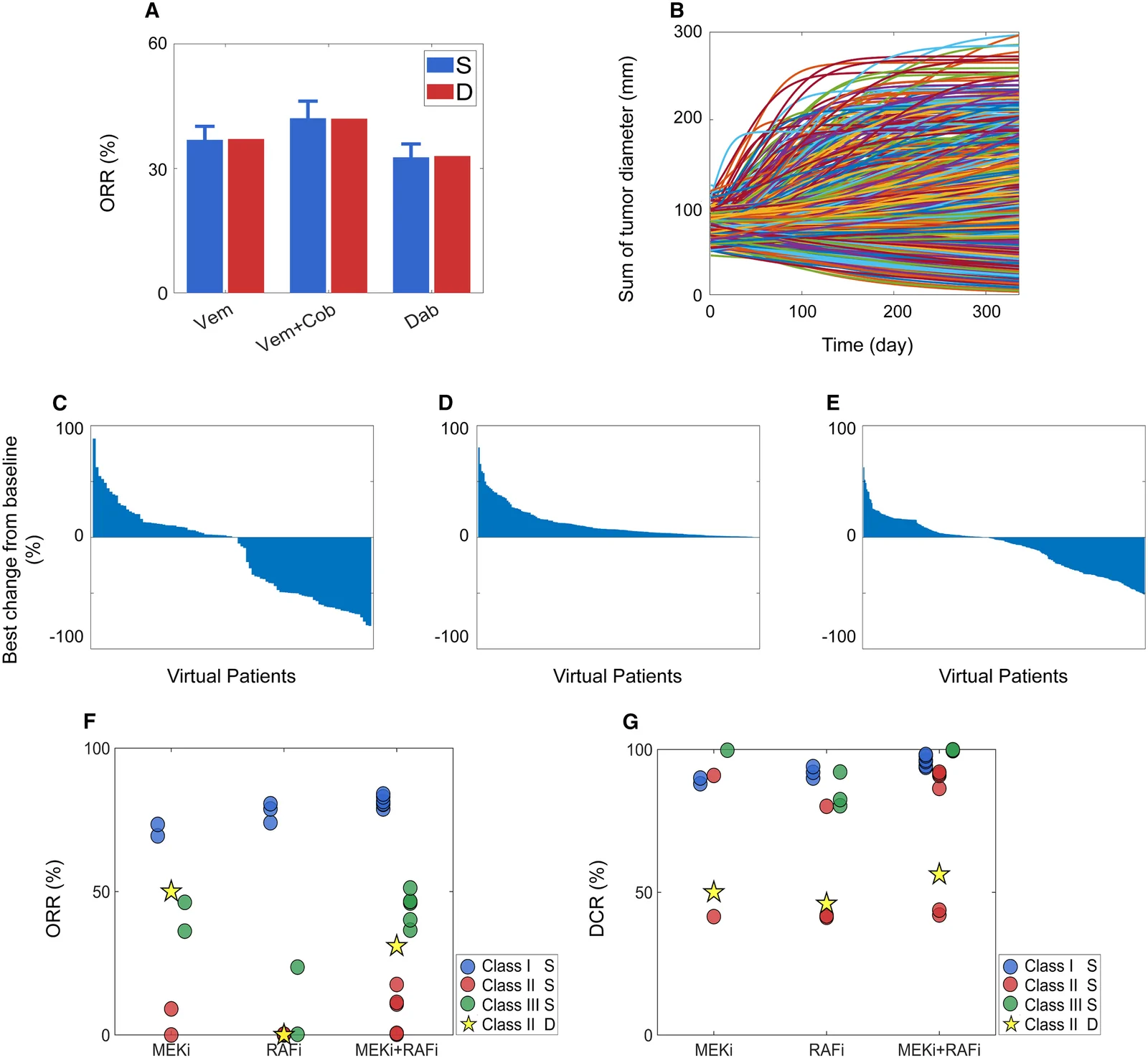
Virtual clinical trials based on the QSP framework revealed mutation class-specific responses to MEKi, RAFi, and their combination across three BRAF-mutant subtypes in NSCLC Using the model-based virtual patient populations, (A) clinical-level response to vemurafenib and dabrafenib (RAFi) and vemurafenib (RAFi) plus cobimetinib (MEKi) combinations was simulated and compared to reported clinical trial data in class I BRAF-mutant NSCLC. (B) Simulated change in sum of tumor diameters in class I virtual patients on vemurafenib (RAFi) monotherapy, depicted in a spider plot. (C–E) Simulated best response to vemurafenib in class I, II, and III virtual patients, depicted in waterfall plots. (F and G) Predicted (using virtual patients and virtual clinical trials) versus clinically reported ORR and DCR values for MEKi, RAFi, and combination therapy across three BRAF mutation classes. S, simulation; D, clinical data.

We also conducted virtual clinical trial analyses to examine the potential efficacy differences of various dosing regimens of targeted inhibitors in BRAF-mutant patients. First, we employed patients with class I BRAF mutations as an example and simulated different combined dosages of trametinib and dabrafenib. At the standard combination dose (trametinib 2 mg qd and dabrafenib 150 mg bid), the model effectively characterized the efficacy results seen in the class I mutant patients in trial NCT01336634.^49^ Model simulations suggested that the optimal ORR is achieved near the approved aforementioned standard dose combination of trametinib and dabrafenib, and further dose escalation of either drug would not lead to improved ORR (Figure 7A). However, in virtual patients with class II BRAF mutations, model simulations suggested that trametinib 2.5 mg qd monotherapy and its combination with dabrafenib 150 mg bid would lead to higher response rates in patients compared to the 2 mg qd regimen (Figures 7B and 7C). Additionally, in virtual patients with class III mutations, simulations also demonstrated a trend of increasing ORR for both vemurafenib and cobimetinib monotherapy as dose increases beyond their respective standard doses (960 mg bid for vemurafenib, 60 mg qd for cobimetinib) approved in the clinic (Figures 7D and 7E), suggesting potential benefits in these patients if they can tolerate higher doses.

**Figure 7:**
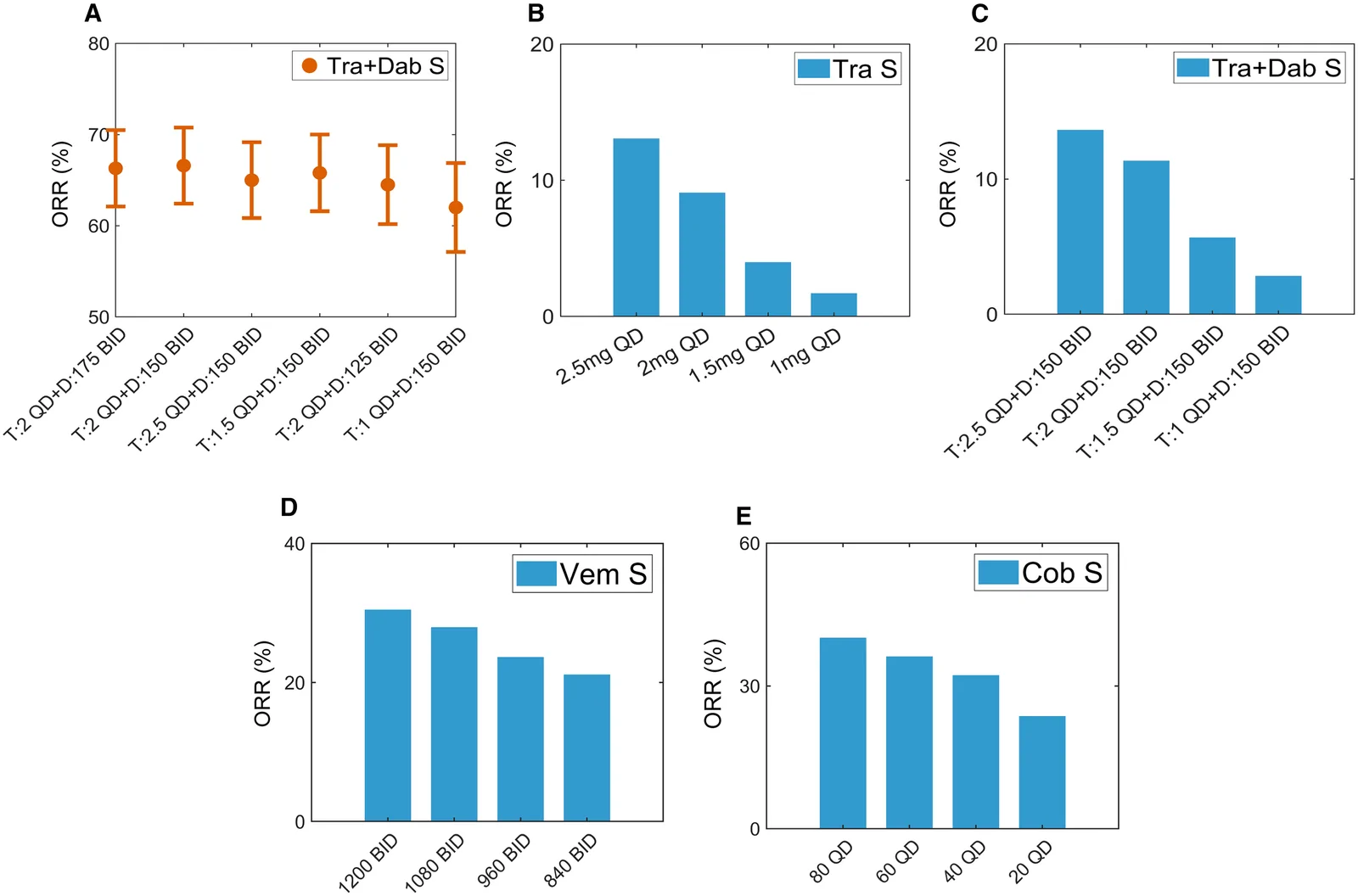
Optimization of clinical drug dosing in class I/II/III BRAF-mutant NSCLC via model-based virtual clinical trials Simulated population-level efficacy in model-based virtual patients of different BRAF mutation classes and under different drug dosages. (A) Simulated response to different trametinib (MEKi) plus dabrafenib (RAFi) regimens in class I BRAF-mutant NSCLC. (B and C) Simulated response to different dosages of trametinib (MEKi) monotherapy and its combination with dabrafenib (RAFi) in class II BRAF-mutant NSCLC. (D and E) Simulated response to different vemurafenib (RAFi) and cobimetinib (MEKi) dosing regimens in class III BRAF-mutant NSCLC.

### High-throughput model-based analysis of optimal pathway-derived combination therapies in different BRAF mutation classes

A global sensitivity analysis was first performed utilizing the partial rank correlation coefficient (PRCC) algorithm in virtual patients with class I–III BRAF-mutant NSCLC to assess the influence of model parameters on the primary outcome measure, tumor size, in the single-agent drug treatment scenario (MEKi trametinib was used here as an example). A summary of the highest-ranked parameters from the analysis is presented in Figures 8A and S9. In short, parameters relating to the signaling activity of protein kinases, including ERK, MEK, AKT, and mTOR, are shown to significantly influence tumor size regression. We then seek to assess the translational potential of different combination regimens in patients with class I-III BRAF-mutant NSCLC by comparatively simulating the population-level clinical response to new combination strategies involving RAFi, MEKi, and ERKi. In class I BRAF-mutant patients, for example, by plugging in the different sets of relevant parameters (derived from *in vitro* signaling and viability, tumor growth inhibition kinetics, and clinical pharmacokinetics) associated with different kinase inhibitors, our simulated clinical response maps indicated generally similar ORRs (around 70%–80%) in patients under several different double combination regimens. Still, the triple combination regimen can yield a modest enhancement in ORRs (∼to 85%) compared to the double combination regimens (Figures 8B and S9). In class II BRAF-mutant NSCLC patients, clinical simulations suggested that trametinib combination with either RAFi (vemurafenib and dabrafenib) or ERKi (ulixertinib and ravoxertinib) can likely demonstrate further improvements in patient tumor shrinkage compared to RAFi monotherapy, and no significant differences were observed among the various double combination regimens (of different drugs) (Figures 8C and S9). Similarly, in class III BRAF-mutant patients, clinical simulations predicted that double combination regimens of MEKi with RAFi or ERKi can lead to superior anti-tumor efficacy compared to all monotherapies. Specifically, the combination of MEKi with ERKi was suggested to exhibit the highest potential in facilitating tumor reduction in these patients (Figures 8D and S9).

**Figure 8:**
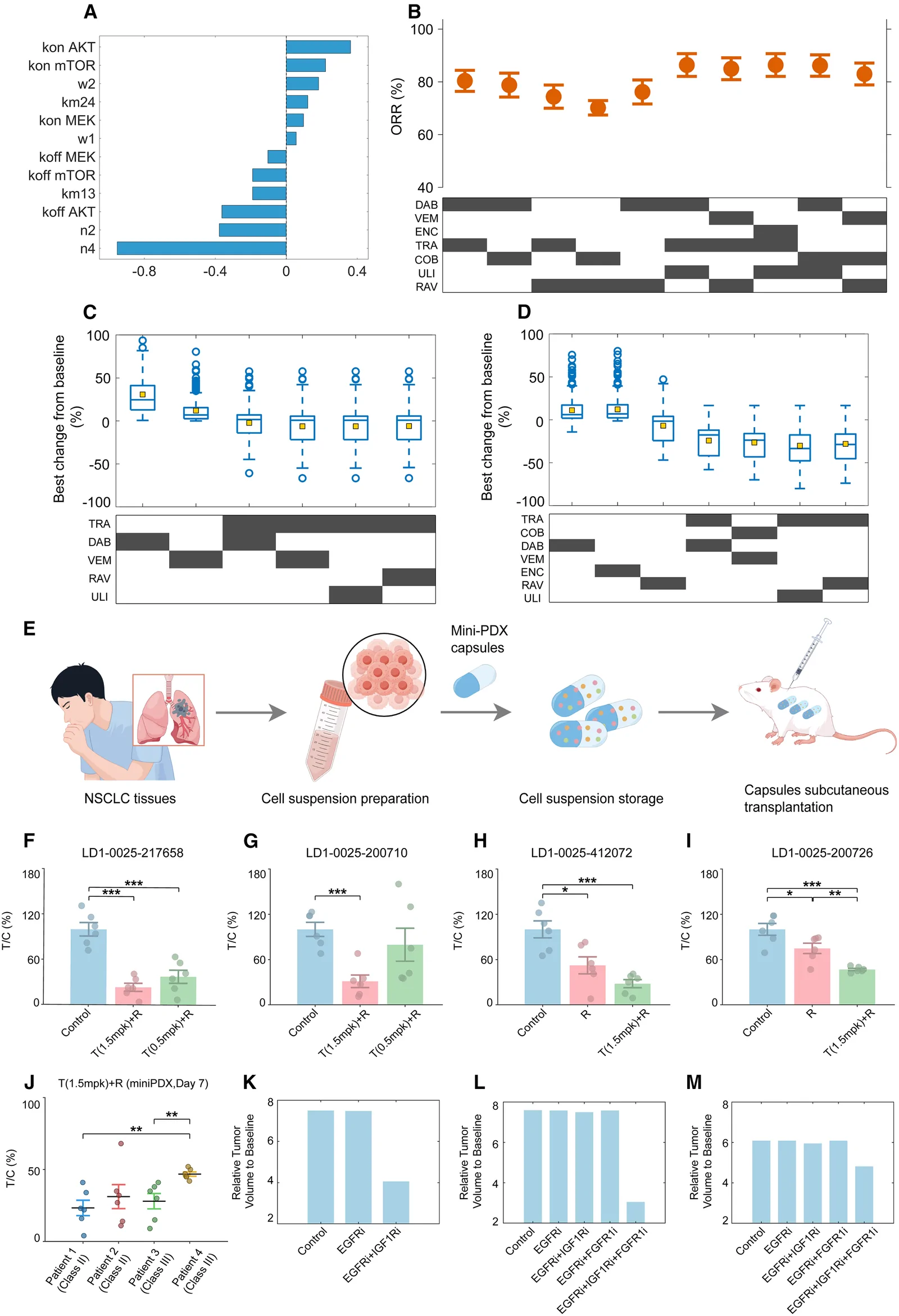
Comprehensive virtual clinical trial exploration and experimental validation of novel combination therapies and co-targeting strategies across class I-III BRAF-mutant NSCLC (A) An exemplar global sensitivity analysis identified key drivers of the anti-tumor response to trametinib in class II BRAF-mutant patients. (B) Using the formulated virtual patient population, responses to RAFi, MEKi, and ERKi combinations in class I BRAF-mutant patients were comparatively simulated. (C and D) Clinical-level response to monotherapy (RAFi, MEKi, or ERKi) and combination therapy (double and triple combinations) in (C) class II and (D) class III BRAF-mutant NSCLC patients were simulated (the yellow squares represent the mean values). (E) Schematic flow diagram of miniPDX *in vivo* experiments. (F–J) *In vivo* MiniPDX anti-tumor efficacy validation of specific combination regimens in two (F–G) class II and (H and I) class III BRAF-mutant experimental models (T/C ratios indicate relative inhibition of tumor growth); (J) summarized day 7 T/C ratios in patient-derived MiniPDX models established from four BRAF-mutant patients (two class II and two class III) treated with combination therapy (T 1.5 mpk qd + R 30 mpk qd); *n* = 6 for each MiniPDX model. (K–M) Simulated fold changes in tumor volume (relative to baseline) under four different treatment conditions: no treatment, partial EGFR inhibition, partial EGFR inhibition combined with IGF1R or FGFR1 inhibition, and concurrent inhibition of EGFR, IGF1R, and FGFR1 in (K) class I, (L) class II, and (M) class III BRAF-mutant NSCLC. Statistical significance was determined by two-sided Student’s *t* test, with ∗*p* < 0.05, ∗∗*p* < 0.01, and ∗∗∗*p* < 0.001. T, trametinib. R, ravoxertinib.

To validate the aforementioned prediction, we conducted original *in vivo* studies with the MiniPDX technology using tumor tissue samples from two NSCLC patients with confirmed class II BRAF mutation and two NSCLC patients with confirmed class III BRAF mutation (see STAR Methods for details). Treating MiniPDX mice with the model-predicted MEKi plus ERKi combination (trametinib plus ravoxertinib) indeed led to significantly reduced tumor growth for both class II and III BRAF-mutant tumors *in vivo* (Figures 8F–8I, denoted by smaller T/C ratios) without affecting animal body weights. In class III BRAF-mutant MiniPDX mice, ravoxertinib alone also showed significant anti-tumor potency (Figures 8H and 8I), which is consistent with our model predictions. Thus, these experimental data (Figures 8F–8J) nicely validated the predictions from our virtual combination trial analyses and confirmed the therapeutic potential of the novel MEKi-ERKi combination in treating class II/III BRAF-mutant NSCLC.

We also investigated the potential impact of modulating EGFR, IGF1R, and FGFR signaling pathways on tumorigenesis and drug resistance by evaluating tumor size changes in patients harboring class I–III BRAF mutations under four treatment conditions: no drug treatment, partial inhibition of the EGFR pathway, partial EGFR pathway inhibition combined with inhibition of either IGF1R or FGFR pathway; and concurrent partial inhibition of EGFR, IGF1R, and FGFR pathways. Pathway inhibition was modeled as the partial blockade of receptor-mediated signaling at the cell surface level (e.g., downregulating ligand-receptor interaction). Simulation results indicated that in patients with class I BRAF-mutant NSCLC, partial inhibition of both EGFR and IGF1R pathways can significantly reduce tumor volume compared to EGFR inhibition alone (Figure 8K). In patients with class II BRAF mutations, any double inhibition (of the three distinct receptors) has limited impact on tumor volume, but triple inhibition can achieve a very significant reduction in tumor volume (Figure 8L). Interestingly, in patients with class III BRAF mutations, double inhibition of the receptor binding/signaling has no impact on tumor volume, and triple pathway inhibition can only result in a modest reduction in tumor volume (Figure 8M). These simulation results provided useful directions for the future understanding of undesirable compensatory pathway activation in class I–III BRAF-mutant NSCLC and highlighted new potential combination approaches to overcome drug resistance.

## Discussion

Therapeutic strategies directed at BRAF mutations remain an active area of investigation in oncology research and drug development. In this study, we developed a multiscale, mechanism-based QSP model that integrates cellular, animal, and patient-level data to support the identification of novel therapeutic combinations and the optimization of treatment regimens for distinct patient subgroups. This QSP modeling framework encompasses complex downstream signaling networks mediated by multiple receptors, including EGFR, IGF1R, and FGFR1, and was iteratively calibrated and validated against extensive preclinical and clinical data. Through simulations and pathway inhibition analyses, we employed the QSP model to predict the potential clinical efficacy of multiple combination regimens targeting different categories of BRAF mutations. The simulation results, together with the *in vivo* experimental validation data presented, can provide critical comparative insights for prioritizing promising therapeutic strategies and offer a theoretical foundation for the design and optimization of subsequent clinical trials.

The current model primarily addresses patients who have not received RAFi/MEKi therapy, but it can also be adapted to simulate patients who develop acquired resistance following treatment. The mechanisms underlying RAFi/MEKi resistance in NSCLC remain incompletely understood, with most evidence derived from studies of BRAF-mutant melanoma. Recognized resistance mechanisms include RTK upregulation, secondary mutations in NRAS and KRAS, and BRAF amplifications.^50^^,^^51^ To overcome these resistance mechanisms, current research and development strategies primarily focus on three approaches: (1) targeting downstream nodes of the MAPK pathway (e.g., ERK),^52^ (2) blocking bypass signaling activation through combinations with SHP2^53^ or PI3K/mTOR inhibitors,^54^ and (3) developing next-generation RAFi (e.g., “paradox breakers,” pan-RAF inhibitors, or RAF dimer inhibitors)^55^^,^^56^^,^^57^ to more effectively suppress BRAF heterodimers and prevent paradoxical activation. Our model is capable of simulating RAFi/MEKi resistance driven by multiple mechanisms and offers a unique framework for comprehensively evaluating combination strategies to overcome this resistance in clinical settings. In addition, some scholars have proposed that class II BRAF mutations can be subdivided into class IIa, which are sensitive to RAFi monotherapy, and class IIb, which are insensitive to RAFi monotherapy.^58^^,^^59^ Although in this study patients with class II BRAF mutations were modeled as RAFi-insensitive—based on clinical trial data from NSCLC patients with such mutations—the model can be adapted to simulate class II BRAF patients who are sensitive to RAFi.

Through our QSP modeling framework, we demonstrated its potential to simulate the population-level efficacy of different therapeutic strategies in NSCLC patients harboring class I, II, or III BRAF mutations. Our model predicted that, among NSCLC patients with class I BRAF mutations, triple therapy yielded a modest improvement in ORR compared with dual therapy. In patients with class II BRAF mutations, combination therapy with MEKi (trametinib) and either RAFi (e.g., vemurafenib or dabrafenib) or ERKi (e.g., ulixertinib or ravoxertinib) was predicted to induce tumor regression. A case report suggested that combined BRAF and MEK inhibition may provide therapeutic benefit in advanced NSCLC patients with class II BRAF mutations.^60^ In a retrospective analysis of 30 NSCLC patients with non-V600E BRAF mutations, Dudnik et al. reported that certain class II mutations associated with high BRAF kinase activity (e.g., G469A and L597R) may respond to BRAFi plus MEKi combination therapy.^61^ These findings are consistent with our model predictions. In patients with class III BRAF mutations, combination therapies of trametinib plus dabrafenib, trametinib plus ulixertinib, trametinib plus ravoxertinib, or cobimetinib plus vemurafenib were predicted to induce tumor regression; among these, the trametinib-ERKi (ulixertinib or ravoxertinib) regimen demonstrated the greatest efficacy. This study further explored the potential therapeutic value of targeting the IGF1R and FGFR pathways to enhance treatment responses. By simulating the dynamic effects of these pathways on tumor volume across different BRAF mutation contexts, we propose that partial inhibition of EGFR combined with IGF1R inhibition may be sufficient to induce substantial tumor regression in class I BRAF mutations, whereas in class II and III BRAF mutations, concurrent inhibition of FGFR1 may provide additional synergistic antitumor effects. These findings suggest that targeting the IGF1R and FGFR1 pathways could represent novel therapeutic strategies for these patient populations. Although these results are based on simulation data and preliminary evidence, they provide important guidance for future investigations. Future work should focus on validating the combined efficacy of IGF1R and FGFR1 inhibitors in preclinical models and clinical trials, as well as exploring their mechanistic basis and potential biomarkers across different BRAF mutation subtypes. It should be mentioned that model-based approaches, such as the QSP framework we developed here, can play increasingly significant roles in informing accurate clinical design and implementation to ensure that patients receive the best dosages of oncology drugs at the bedside, especially as the Project Optimus initiative becomes more and more important throughout the clinical development of new oncology drugs.^62^

### Limitations of the study

First, while the QSP model predicts that triple blockade (EGFR + IGF1R + FGFR inhibitors) may overcome resistance in class II/III BRAF-mutant NSCLC, we did not evaluate the safety of this combination. Based on known adverse effects of these inhibitors, the triple regimen is expected to cause a range of toxicities, including skin rash, diarrhea, mucositis, hyperglycemia, and metabolic disturbances. Concurrent inhibition may produce additive or synergistic toxicities, potentially limiting clinical feasibility. Our current QSP framework focuses on tumor signaling pathways and does not include normal tissue physiology or off-target toxicity mechanisms. Future work should integrate quantitative systems toxicology models or combine preclinical toxicity studies and adaptive phase I dose-finding trials to assess the therapeutic window of this triple combination. Second, our model does not account for plasma protein binding and does not include a full physiologically-based pharmacokinetic (PBPK) model with a dedicated tumor compartment. The drugs investigated are small molecule kinase inhibitors, for which intratumoral concentration data are rarely available. Given the lack of such data and the comparative nature of our study (focusing on relative efficacy across mutation classes), we adopted practical simplifications: assuming proportional intratumoral exposure with a fixed tumor partition coefficient and omitting the unbound fraction. These simplifications are reasonable for our comparative purpose, as the relative rankings across mutation classes and drug combinations are unlikely to be significantly affected. Future work with richer intratumoral PK data could incorporate a minimal PBPK model or include the unbound fraction explicitly. Third, our model does not account for inter-patient PK variability. We focus mainly on the impact of pharmacodynamic variability and employ fixed PK parameters and a fixed tumor partition coefficient as a simplification to compare the relative efficacy across mutation classes and drug combinations. This simplification is widely used in QSP modeling for comparative predictions and is reasonable for our study purpose. Future work could incorporate population PK or PBPK models to more precisely capture individual differences in exposure and tumor penetration. Fourth, due to limited published data for class II/III BRAF-mutant NSCLC, our validation is based primarily on ORR/DCR derived from published waterfall plots (tumor shrinkage distributions), which were also used to construct our virtual patient population. The published literature lacks sufficient progression-free survival, drug resistance, and exposure-response data for these subgroups. Richer clinical data will enable more comprehensive validation in the future.

## Resource availability

### Lead contact

Requests for further information and resources should be directed to and will be fulfilled by the lead contact, Chen Zhao (chenzhao22@njmu.edu.cn).

### Materials availability

This study did not generate new, unique reagents.

### Data and code availability

• Data reported in this paper will be shared by the lead contact upon request.
• Runnable MATLAB model code for the exemplar simulation scenarios (covering *in vitro* signaling dynamics, *in vivo* tumor growth inhibition, mouse and human pharmacokinetics, and virtual clinical trials) presented in this study has been deposited in the Zenodo open-access repository and is freely available for download at https://doi.org/10.5281/zenodo.20852473.
• Any additional information required to reanalyze the data reported in this paper is available from the lead contact upon request.

## Acknowledgments

This work was supported by CAST Young Scientist Program #YESS20210160, Jiangsu Provincial Natural Science Major Project #BK20222008, and #BM2023004.

## Author contributions

J.H., C.Z., and G.Z. conceptualized and supervised the study. J.H. and Q.R. implemented the computational model and performed model simulations and analyses. Z.B. and D.W. helped with the PDO testing and experimental data analyses. Z.L. and G.L. helped with the modeling and data analyses. J.H., Q.R., C.Z., and G.Z. wrote the first draft, and all authors contributed to the review and editing of the manuscript.

## Declaration of interests

The authors have declared that no competing interests exist.

## STAR★Methods

### Key resources table

**Table:** 

REAGENT or RESOURCE	SOURCE	IDENTIFIER
**Software and algorithms**		

MATLAB code for QSP model implementation and simulation	This paper [Zenodo]	https://doi.org/10.5281/zenodo.20852473
MATLAB Simbiology	MathWorks	R2020a, https://www.mathworks.com/
ImageJ	NIH	RRID:SCR_003070

### Experimental model and study participant details

#### Mice

6 to 8-week-old female BALB/c nude mice were used for animal experiments. All animal experiments and procedures were approved by the Institutional Animal Care and Use Committee of Shanghai Liwen Biotech Co., Ltd (IACUC Approval Code: LWSW-PD42-DT02) and performed in an SPF facility. The procedures were conducted following AAALAC guidelines.

BRAF-mutant NSCLC miniPDX models were generated by Shanghai LIDE Biotechnology Co., Ltd. (Shanghai, China) with informed consent obtained from four NSCLC patients: LD1-0025-200710-FP1+2, a 52-year-old Asian male with right primary bronchogenic lung squamous cell carcinoma (c-T2N2M1b, stage IV, PS score 1) harboring Class II BRAF mutation; LD1-0025-217658-FP1+1, a 54-year-old Asian male with lung adenocarcinoma (c-T2N3M1c, stage IVB1, EGFR/ALK/ROS1 negative, prior one cycle of pemetrexed plus carboplatin) harboring Class II BRAF mutation; LD1-0025-200726-FP4+1, a 68-year-old Asian male with poorly differentiated lung adenocarcinoma (treatment-naive, left upper lung biopsy) harboring Class III BRAF mutation; LD1-0025-412072-FP2+2D, a 51-year-old Asian male with poorly differentiated lung adenocarcinoma with neuroendocrine marker expression (prior right middle lobe resection in 2017, recurrence with intracranial and bone metastasis, prior osimertinib treatment with progression, liver metastasis biopsy for modeling) harboring Class III BRAF mutation. The research protocols were approved by the Ethics Committee of Shanghai Chest Hospital (Approval No. SHLD-SCA-20161014001–111; Acceptance No. KS(Y)1655) for patients LD1-0025-200710-FP1+2, LD1-0025-217658-FP1+1 and LD1-0025-200726-FP4+1, and by the China Ethics Committee of Registering Clinical Trials (Approval No. SHLW-SCA-20191101001–4; Review No. ChiECRCT20190201) for patient LD1-0025-412072-FP2+2D. All procedures were conducted in accordance with the Declaration of Helsinki.

### Method details

#### Model formulation, calibration and in silico pathophysiological simulation

The comprehensive QSP model was formulated using ordinary differential equations (ODEs) based on mass action kinetics and Hill-type reaction principles, encompassing about 79 species and 70 equations (including drug PK, see Figure S10). Intracellular signaling processes within the EGFR, FGFR1, and IGF1R pathways, including receptor-ligand binding, degradation, phosphorylation, and downstream kinase activation, are predominantly characterized by mass action kinetics. The model includes three categories of protein kinase inhibitors: MEKi (trametinib and cobimetinib), RAFi (vemurafenib, dabrafenib, and encorafenib), and ERKi (ulixertinib and ravoxertinib). All therapeutics come with their respective PK modules (two-compartment) under the premise that drug exposure within the tumor is proportional to that in plasma.

Preclinical data from cell and animal studies were used selectively based on expected cross-species conservation. Parameters that are mechanistically conserved—namely drug-target binding affinities, reaction rate constants for pathway signaling (phosphorylation, dephosphorylation, degradation), and pathway topology—were derived from *in vitro* or human cell line data and directly translated to the human model. PK parameters were calibrated separately for mouse and human using species-specific plasma concentration data, with no cross-species scaling applied. In contrast, tumor growth and death rates were not extrapolated from animals; instead, the net tumor growth rate was calculated directly from clinical patient volume doubling time data in NSCLC to ensure the model reflects human disease kinetics.

To simulate a virtual patient population, inter-patient variability was incorporated for several key parameters: initial membrane receptor levels (EGFR, FGFR1, IGF1R), initial tumor cell number (reflecting different tumor burdens), and the differential modulation of tumor growth by BRAF mutation class (Class I, II, and III), each having distinct effects on MAPK pathway activity and growth dependence. These variable parameters were sampled from literature- or clinical data-informed ranges, allowing the model to capture response heterogeneity. By contrast, across the virtual patient population, PK parameters and the tumor partition coefficient were kept fixed (tumor drug concentration assumed proportional to plasma) as a simplification to focus on comparing relative efficacy across mutation classes and drug combinations.

In model calibration, we initially employed experimental data at the cellular level (*in vitro* experiments), encompassing measures of signal transduction and drug-induced alterations in cell viability. We utilized A549, an NSCLC cell line characterized by relatively normal EGFR, FGFR1, IGF1R, and BRAF status, as the reference cell line. Quantitative literature data indicated that the receptor counts per A549 cell were approximately 158,835 for EGFR, 14,718 for FGFR1, and 8,710 for IGF1R.^63^ Given that FGFR1 can induce spontaneous activation by autophosphorylation without ligands, we permitted all model species to attain their respective steady states, subsequently utilizing these states as initial conditions for simulating additional changes and drug treatments. In calibrating the model at the *in vivo* level, we adjusted the tumor doubling time by aligning it with experimental tumor growth data obtained under control conditions (no treatment). Subsequently, mouse PK data were incorporated to establish a connection between drug administration and drug-induced tumor growth inhibition *in vivo*. A list of model species, equations and parameters are provided in . Runnable MATLAB model code is available at Zenodo repository (https://doi.org/10.5281/zenodo.20852473).

In the construction of virtual patients and virtual patient populations, it is assumed that tumors exhibit spherical growth, with tumor diameter measured every 6–8 weeks and responses evaluated according to the RECIST v1.1 criteria. Monte Carlo sampling was performed using log-normal distributions to generate a virtual patient population reflecting inter-individual physiological variability. For each BRAF mutation class (Class I, II, and III), 5,000 virtual patients were generated. Key parameters were randomized within biologically plausible ranges: BRAF-driven MEK phosphorylation activity based on reported MAPK pathway variability across Class I-III BRAF-mutant NSCLC patients^26^; tumor cell growth rate derived from literature on NSCLC doubling times^64^; tumor cell death rate fitted to NSCLC clinical data after fixing the growth rate; and initial tumor diameter based on NSCLC clinical data.^65^ Baseline values for these parameters (see Table S1) were obtained from literature or model calibration. The standard deviation σ was set to 0.5 or 1, and the 95% confidence range was calculated as exp(ln(baseline) ± 1.96×σ). To further capture physiological individual differences, additional parameters such as the activation degree of BRAF on MEK phosphorylation were also allowed to vary within reasonable numerical ranges during Monte Carlo sampling. Virtual patients with zero or negative net tumor growth rate (net growth rate ≤0) or with tumor doubling times inconsistent with clinical data from BRAF-mutant patients^66^^,^^67^ were excluded. The remaining virtual patients were then weighted using the inclusion probability method established by,^68^ with weights assigned based on clinical objective response rates. The final virtual patient population was selected to align its efficacy distribution with that of the previously mentioned target drug clinical study. Virtual clinical trials are conducted on a virtual patient population with various classes of BRAF mutations in the NSCLC setting to simulate the patient response to various treatment scenarios.

The ImageJ software (NIH, Bethesda, USA) was utilized for the quantification analysis of published western blotting data. The experimental data values were standardized to their corresponding maximum or control levels. Cell survival rate fitting involved normalizing cell growth data obtained under drug treatment situations to that of untreated conditions during the same time period, resulting in simulated curves for cell survival rates. All model reactions were executed in the MATLAB Simbiology Toolbox (MathWorks, Natick, MA, USA) and simulated utilizing the ode15s solver. Parameter values were established using experimental data, such as binding and dissociation rates, as well as protein half-life, or derived through data fitting strategies. The patternsearch function within MATLAB’s Global Optimization Toolbox was employed for parameter estimation and optimization purposes.

#### Global sensitivity analyses

The partial rank correlation coefficient (PRCC) algorithm was executed following the methodology established by Marino et al.^69^ The algorithm was configured with 5,000 iterations and employed Latin hypercube sampling for each input condition. The parameter value range chosen for the PRCC calculation was established at a 5% increase and decrease relative to the baseline. The sum of tumor diameter on day 336 was the primary output for all PRCC calculations. Parameters significantly correlated with model outputs were analyzed for each type of BRAF mutation in the context of trametinib monotherapy (e.g., PRCC values exceeding 0.05 or falling below −0.05, accompanied by statistical significance).

#### MiniPDX drug sensitivity assay

Four patient-derived xenografts (PDX) were used: two with Class II BRAF mutations (LD1-0025-200710-FP1+2, LD1-0025-217658-FP1+1) and two with Class III BRAF mutations (LD1-0025-200726-FP4+1, LD1-0025-412072-FP2+2D). Cryopreserved tumors were thawed, expanded subcutaneously in female NKG mice, and processed into single-cell suspensions upon reaching 500–800 mm^3^. Viable cells (3×10^3^ per capsule) were seeded into MiniPDX capsules and implanted into female BALB/c nude mice. All procedures were approved by the Institutional Animal Care and Use Committee (IACUC Approval Code: LWSW-PD42-DT02) and performed in an SPF facility. For each model, mice received daily oral treatments for 7 days from Day 0:

Class II models: Control, trametinib (1.5 mg/kg) + ravoxertinib (30 mg/kg), trametinib (0.5 mg/kg) + ravoxertinib (30 mg/kg).

Class III models: Control, ravoxertinib (30 mg/kg), trametinib (1.5 mg/kg) + ravoxertinib (30 mg/kg).

Drugs were formulated in 0.5% HPMC/0.2% Tween 80. Body weight was monitored daily, and relative change (RCBW) was calculated as: RCBW (%) = [(BWᵢ − BW_0_)/BW_0_] × 100. Capsules were harvested on Day 7. Cell viability was measured using the CellTiter-Glo assay. The relative proliferation rate was calculated as (T/C) × 100%, where T and C are the mean viabilities of treatment and control groups (six capsules per group). Lower values indicate greater inhibition.

### Quantification and statistical analysis

This study utilized R-square (R^2^) and Mean Absolute Percentage Error (MAPE) as quantitative metrics to assess the goodness of fit between model predictions and experimental data on *in vivo* antitumor activity. R^2^ varies between 0 and 1, with values approaching 0 signifying reduced prediction accuracy and values nearing 1 signifying enhanced prediction accuracy. A MAPE of less than 20% is typically deemed acceptable.^70^ For MiniPDX experiments, statistical comparisons were made using two-sided unpaired Student’s *t* test (*n* = 6 per model). Statistical significance is denoted as ∗*p* < 0.05, ∗∗*p* < 0.01, ∗∗∗*p* < 0.001.

## Supplemental information

Document S1. Figures S1–S10

Table S1. Details of model description, species, parameters, and equations, related to Figure 1
