# Supplementary material for: Quantitative systems pharmacology modeling of class I–III BRAF-mutant NSCLC enables subgroup treatment analysis and combination therapy design
Source: iScience. 2026 Oct 13;29(11):117854. doi: 10.1016/j.isci.2026.117854 (PMC13682387; doi:10.1016/j.isci.2026.117854)
Supplement: Supplementary file 1 [file mmc1.pdf]

iScience, Volume *29*

Supplemental information

Quantitative systems pharmacology modeling of class I–III BRAF-mutant NSCLC enables subgroup treatment analysis and combination therapy design

Jingjing Hu, Qi Rao, Zichun Li, Geli Li, Zhenle Bi, Danyi Wen, Chen Zhao, and Guohua Zhou

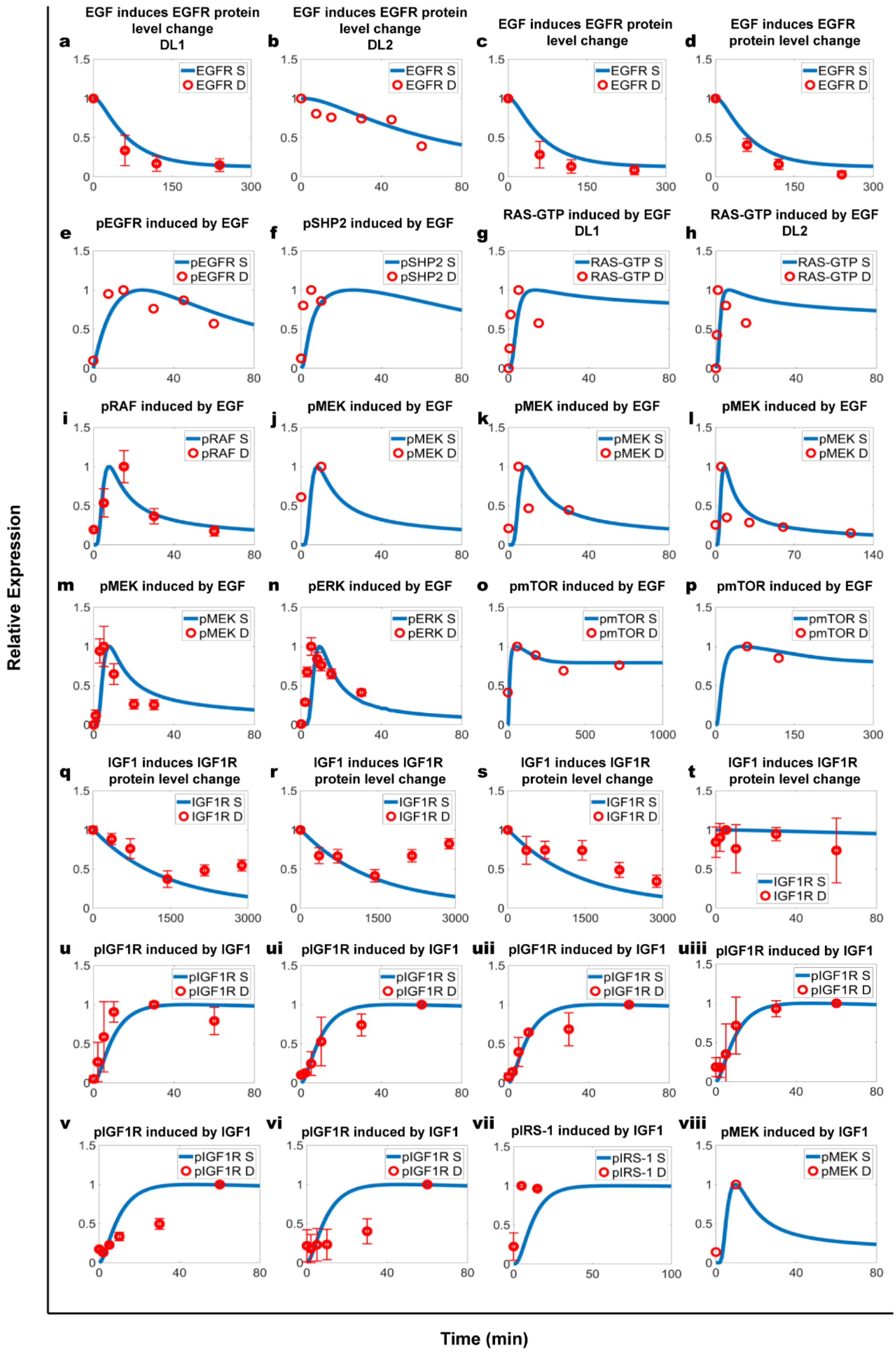

a EGF induces EGFR protein level change DL1
b EGF induces EGFR protein level change DL2
c EGF induces EGFR protein level change
d EGF induces EGFR protein level change
e pEGFR induced by EGF
f pSHP2 induced by EGF
g RAS-GTP induced by EGF DL1
h RAS-GTP induced by EGF DL2
i pRAF induced by EGF
j pMEK induced by EGF
k pMEK induced by EGF
l pMEK induced by EGF
m pMEK induced by EGF
n pERK induced by EGF
o pmTOR induced by EGF
p pmTOR induced by EGF
q IGF1 induces IGF1R protein level change
r IGF1 induces IGF1R protein level change
s IGF1 induces IGF1R protein level change
t IGF1 induces IGF1R protein level change
u pIGF1R induced by IGF1
ui pIGF1R induced by IGF1
uii pIGF1R induced by IGF1
uiii pIGF1R induced by IGF1
v pIGF1R induced by IGF1
vi pIGF1R induced by IGF1
vii pIRS-1 induced by IGF1
viii pMEK induced by IGF1
EGFR S
EGFR D
pEGFR S
pEGFR D
pSHP2 S
pSHP2 D
RAS-GTP S
RAS-GTP D
pRAF S
pRAF D
pMEK S
pMEK D
pERK S
pERK D
pmTOR S
pmTOR D
IGF1R S
IGF1R D
pIGF1R S
pIGF1R D
pIRS-1 S
pIRS-1 D
Relative Expression
Time (min)

Supplementary Fig S1. Additional model calibration using pathway signal transduction data (Part 1), related to Fig. 2. In cancer cells with normal BRAF, **(a-d)** EGF (**a**-20 ng/mL, **b**-100 ng/mL,**c**-20 ng/mL,**d**-20 ng/mL) induces dimerization, internalization and degradation of EGFR, along with **(e)** phosphorylating activation of EGFR (100 ng/mL)[1, 2]. **(f)** EGF (20 nM) induces downstream activation of SHP2[3]. **(g-h)** EGF (**g**-0.1 nM, **h**-1nM) induces downstream activation of RAS-GTP[3]. **(i)** EGF (100 ng/mL) induces downstream activation of RAF[4]. **(j-m)** EGF induces downstream activation of MEK (**j**-20 ng/mL, **k**-10 ng/mL,**l**-30 ng/mL,**m**-20 nM)[3, 5-7]. **(n)** EGF (1 nM) induces downstream activation of ERK [8]. **(o-p)** EGF (**o**-25 ng/mL, **p**-10 ng/mL) induces downstream activation of ERK [9, 10]. For the IGF1R pathway, **(q-t)** IGF1 (50 ng/mL) induces dimerization, internalization and degradation of IGF1R, along with **(u-vi)** phosphorylating activation of IGF1R[11, 12]. **(vii)** IGF1 (40 ng/mL) induces downstream activation of IRS-1 [13]. **(viii)** IGF1 (100 ng/mL) induces downstream activation of MEK[14]. S, simulation; D, experimental *in vitro* data; DL, dose level. Y-axes are relative expression levels (normalized to the respective maximum values).

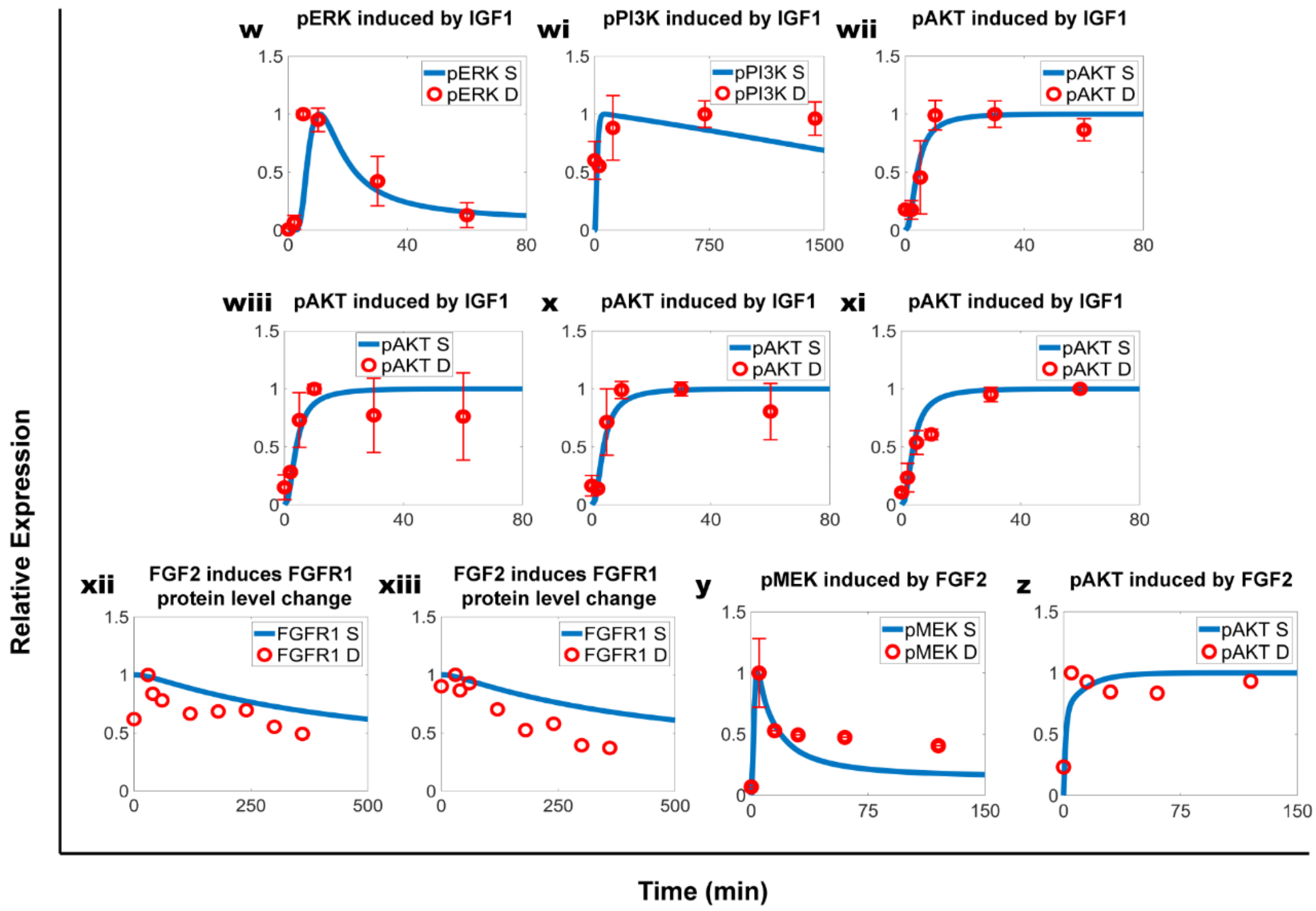

Supplementary Fig S2. Additional model calibration using pathway signal transduction data (Part 2), related to Fig. 2. In cancer cells with normal BRAF, **(w)** IGF1 (50 ng/mL) induces downstream activation of ERK [12]. **(wi)** IGF1 (100 ng/mL) induces downstream activation of PI3K[15]. **(wii-xi)** IGF1 (50 ng/mL) induces downstream activation of AKT [11]. For the FGFR1 pathway, **(xii-xiii)** FGF2 induces dimerization, internalization and degradation of FGFR1 (**xii**- 20 ng/mL, **xiii**-25 ng/mL) [16, 17]. **(y)** FGF2 (1 nM) induces downstream activation of MEK[18]. **(z)** FGF2 (10 ng/mL) induces downstream activation of AKT [19]. S, simulation; D, experimental *in vitro* data; DL, dose level. Y-axes are relative expression levels (normalized to the respective maximum values).

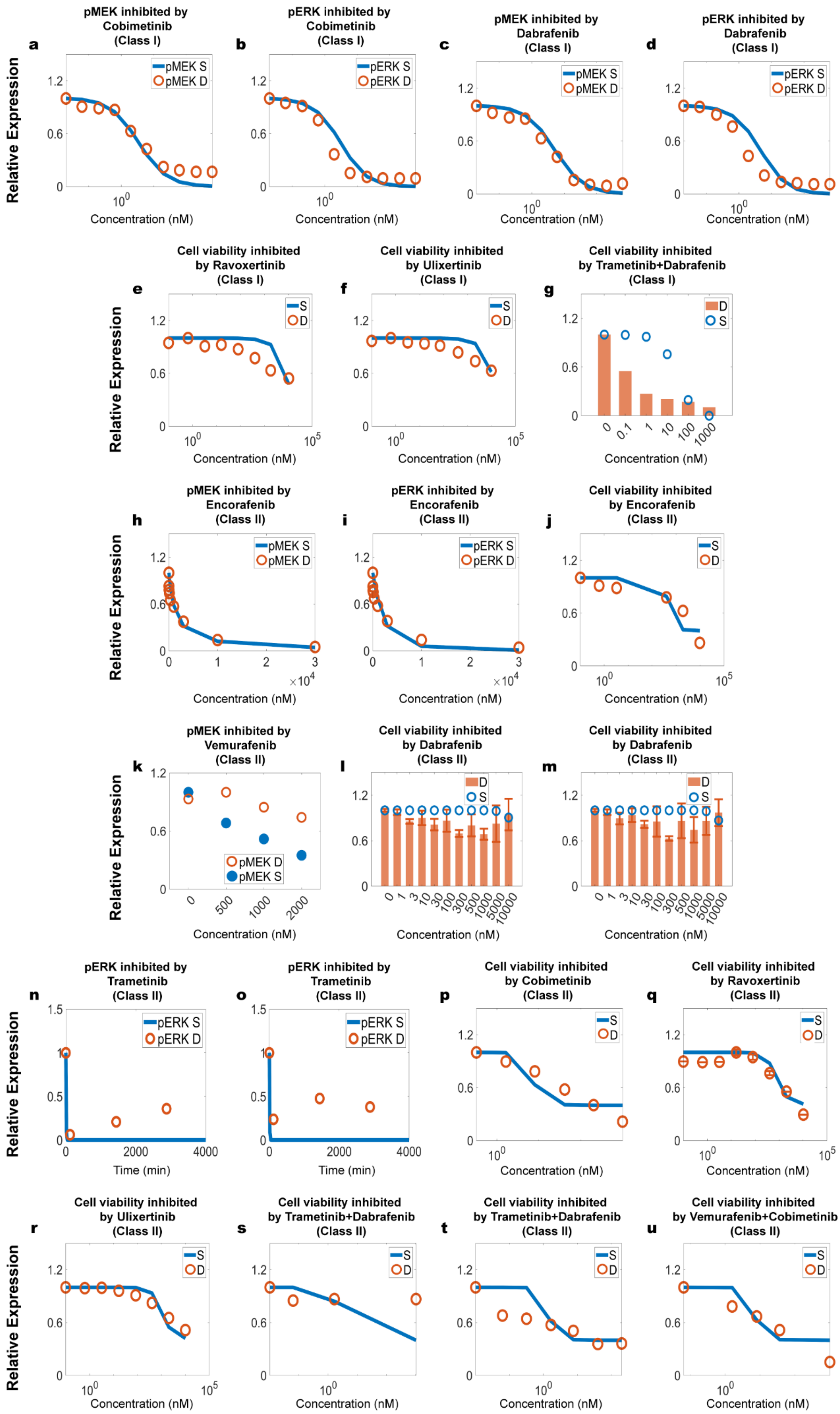
a
pMEK inhibited by Cobimetinib (Class I)
pMEK S
pMEK D
b
pERK inhibited by Cobimetinib (Class I)
pERK S
pERK D
c
pMEK inhibited by Dabrafenib (Class I)
pMEK S
pMEK D
d
pERK inhibited by Dabrafenib (Class I)
pERK S
pERK D
e
Cell viability inhibited by Ravoxertinib (Class I)
f
Cell viability inhibited by Ulixertinib (Class I)
g
Cell viability inhibited by Trametinib+Dabrafenib (Class I)
h
pMEK inhibited by Encorafenib (Class II)
pMEK S
pMEK D
i
pERK inhibited by Encorafenib (Class II)
pERK S
pERK D
j
Cell viability inhibited by Encorafenib (Class II)
k
pMEK inhibited by Vemurafenib (Class II)
pMEK D
pMEK S
l
Cell viability inhibited by Dabrafenib (Class II)
m
Cell viability inhibited by Dabrafenib (Class II)
n
pERK inhibited by Trametinib (Class II)
pERK S
pERK D
o
pERK inhibited by Trametinib (Class II)
pERK S
pERK D
p
Cell viability inhibited by Cobimetinib (Class II)
q
Cell viability inhibited by Ravoxertinib (Class II)
r
Cell viability inhibited by Ulixertinib (Class II)
s
Cell viability inhibited by Trametinib+Dabrafenib (Class II)
t
Cell viability inhibited by Trametinib+Dabrafenib (Class II)
u
Cell viability inhibited by Vemurafenib+Cobimetinib (Class II)
S
D
Relative Expression
Concentration (nM)
Time (min)

Supplementary Fig S3. Additional model calibration using cell viability data under various drug interventions (Part 1), related to Figs. 3, 4 and 5. In Class I BRAF-mutant cell lines, cobimetinib (MEKi) induces dose-dependent inhibition of **(a)** MEK and **(b)** ERK activation[20]. Dabrafenib (RAFi) induces dose-dependent inhibition of **(c)** MEK and **(d)** ERK activation [20]. **(e)** Ravoxertinib (ERKi), **(f)** ulixertinib (ERKi), and **(g)** the trametinib (MEKi) plus dabrafenib (RAFi) combination all inhibit cell viability in dose-dependent manners[21]. In Class II BRAF-mutant cell lines, encorafenib (RAFi) induces dose-dependent inhibition of **(h)** MEK, **(i)** ERK activation, and **(j)** cell viability [21, 22]. Vemurafenib (RAFi) induces dose-dependent inhibition of **(k)** MEK activation [23]. Dabrafenib (RAFi) induces dose-dependent inhibition of **(l-m)** cell viability[24]. Trametinib (MEKi) inhibits **(n)** MEK and **(o)** ERK activation in a dose-dependent manner. **(p)** Cobimetinib (MEKi), **(q)** ravoxertinib (ERKi), (**r**) ulixertinib (ERKi), **(s-t)** trametinib (MEKi) plus dabrafenib (RAFi) combination and **(u)** the vemurafenib (RAFi) plus cobimetinib (MEKi) combination all inhibit cell viability in dose-dependent manners[21, 24]. S, simulation; D, experimental *in vitro* data. Y-axes are relative expression levels (normalized to the no treatment condition).

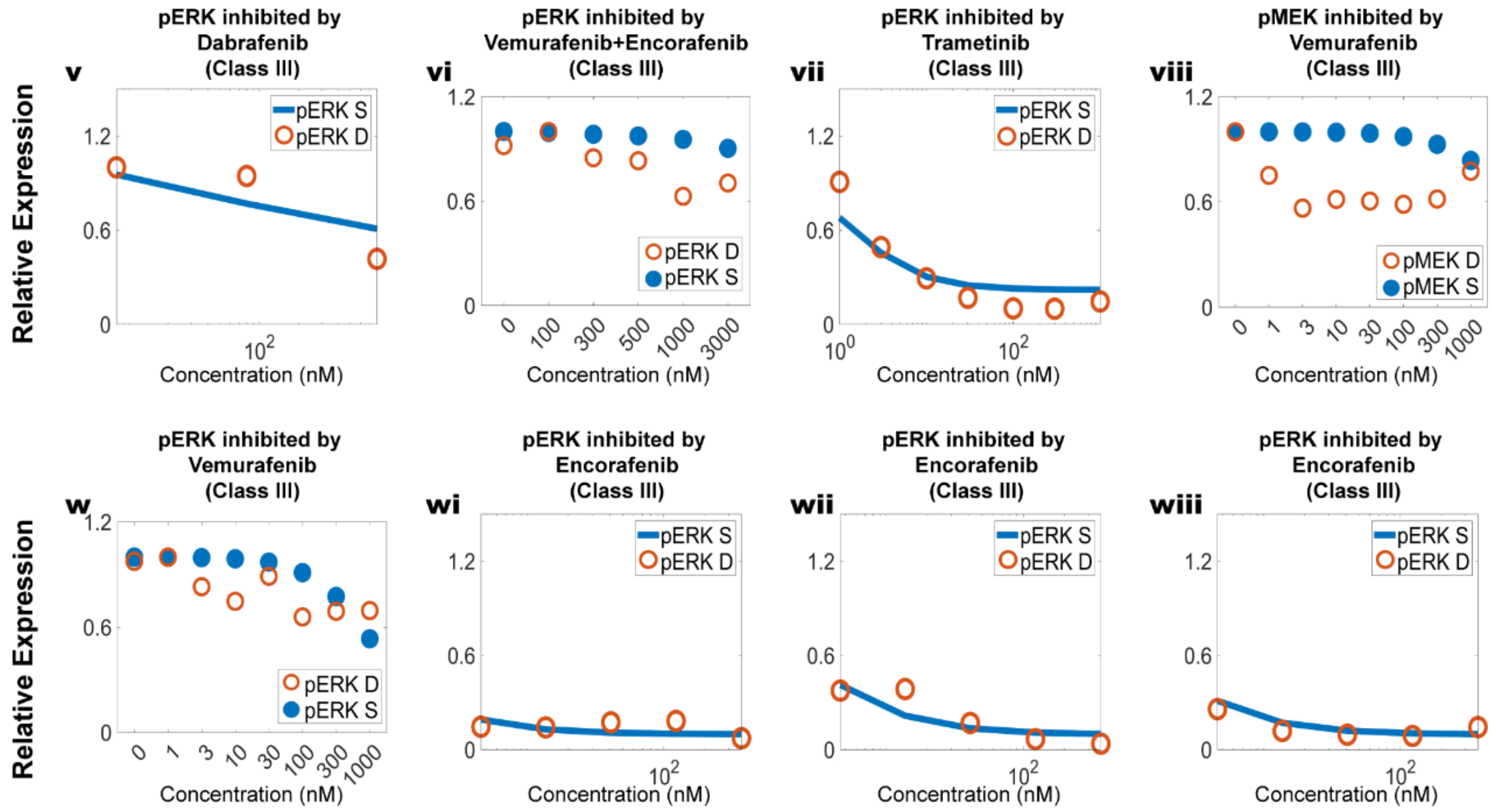

Supplementary Fig S4. Additional model calibration using cell viability data under various drug interventions (Part 2), related to Figs. 3, 4 and 5. In Class III BRAF-mutant cell lines, **(v)** dabrafenib (RAFi) and **(vi)** the vemurafenib (RAFi) plus encorafenib (RAFi) induces dose-dependent inhibition of MEK activation [25, 26]. In Class III BRAF-mutant cancer cell lines harboring a co-occurring RAS mutation, trametinib (MEKi) induces dose-dependent inhibition of **(vii)** ERK activation[27]. Vemurafenib (RAFi) induces dose-dependent inhibition of **(viii)** MEK and **(w)** ERK activation[27, 28]. Encorafenib (RAFi) induces dose-dependent inhibition of **(wi)** ERK activation[28]. In Class III BRAF-mutant cancer cell lines harboring a co-occurring NF1 loss, encorafenib (RAFi) induces dose-dependent inhibition of **(wii-wiii)** ERK activation[28]. S, simulation; D, experimental *in vitro* data. Y-axes are relative expression levels (normalized to the no treatment condition).

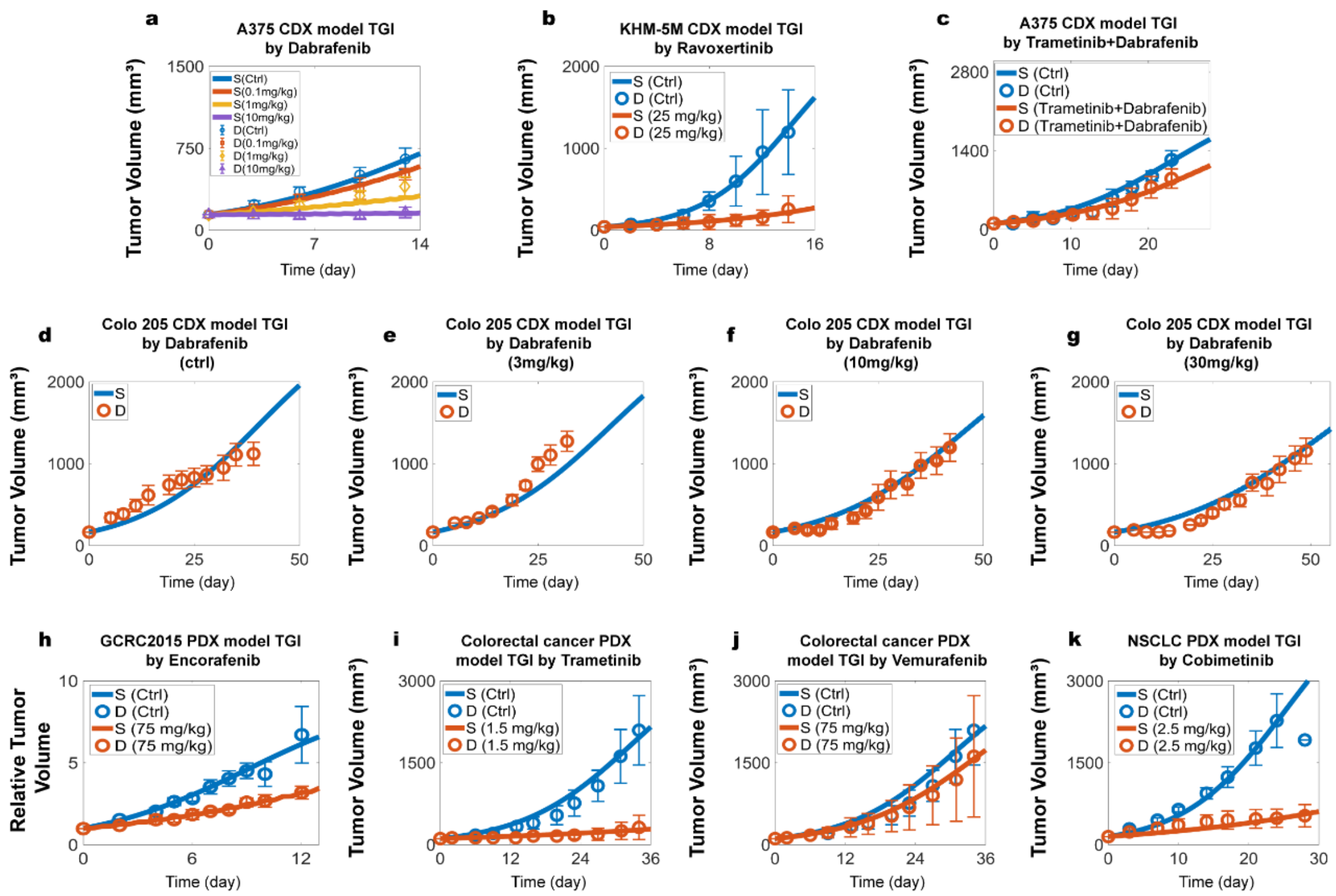

Supplementary Fig S5. Additional model calibration using *in vivo* tumor growth inhibition data in response to various drugs, related to Fig. 5. The QSP model accurately recapitulates the reported *in vivo* antitumor efficacy of BRAF pathway inhibitors in both cell-derived xenograft (CDX) and patient-derived xenograft (PDX) models. **(a-g)** CDX model responses to: **(a)** dabrafenib (RAFi); **(b)** ravoxertinib (ERKi); **(c)** dabrafenib (RAFi) plus trametinib (MEKi) combination; **(d-g)** dabrafenib (RAFi) [29-32]. **(h-k)** PDX model responses to: **(h)** encorafenib (RAFi); **(i)** trametinib (MEKi); **(j)** vemurafenib (RAFi); **(k)** cobimetinib (MEKi)[27, 33, 34]. S, simulation; D, experimental *in vivo* data.

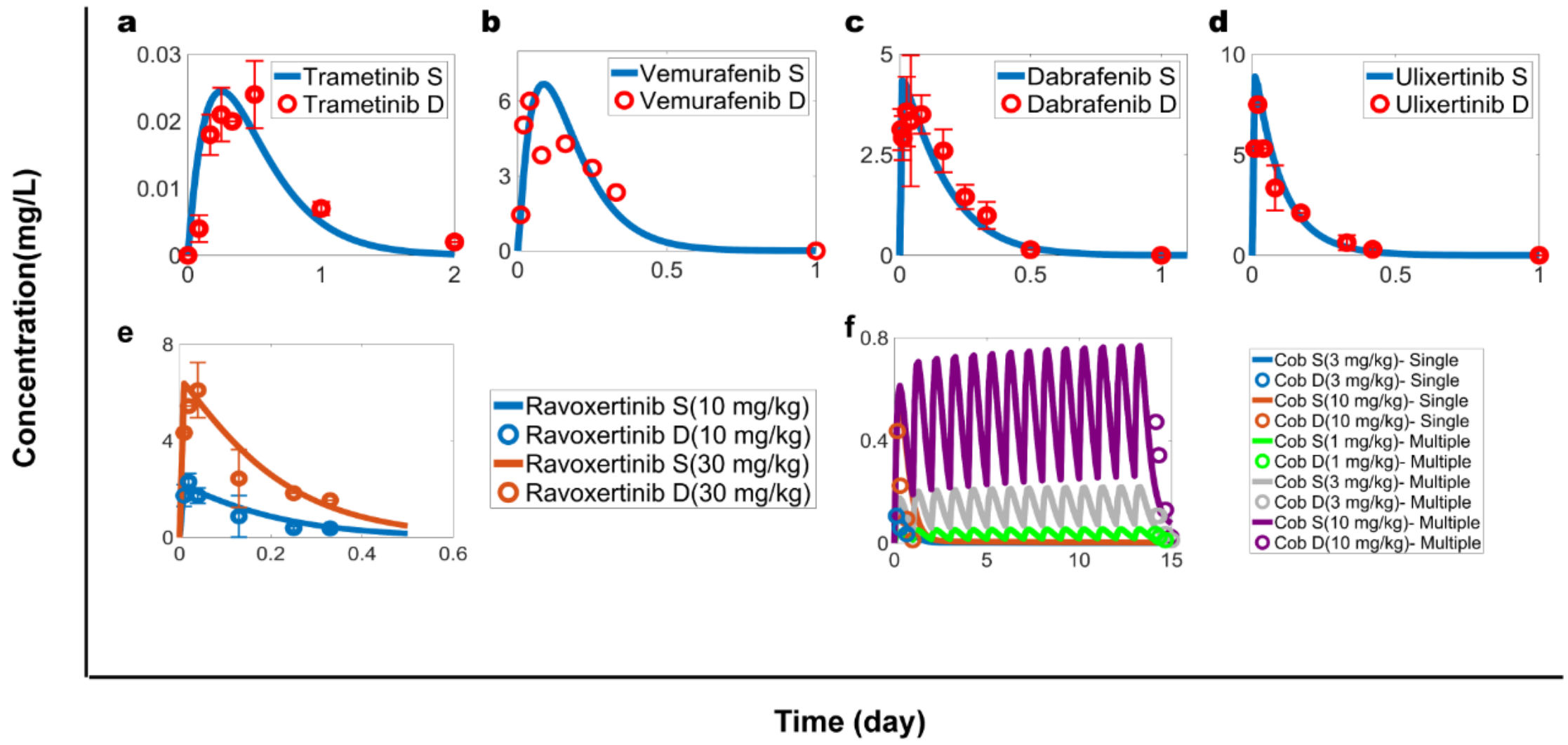

Supplementary Fig S6. QSP submodules describing PK of various drugs in mouse, related to Fig. 5. In the QSP model, we incorporated PK submodules of various drugs in mouse that are calibrated against published data, including: **(a)** plasma concentrations of trametinib following a single oral dose of 5 mg/kg [35]; **(b)** plasma concentrations of vemurafenib following a single oral dose of 25 mg/kg[36]; **(c)** plasma concentrations of dabrafenib following a single oral dose of 10 mg/kg[37]; **(d)** plasma concentrations of ulixertinib following a single oral dose of 10 mg/kg[38]; **(e)** plasma concentrations of ravoxertinib following a single oral dose of 10 or 30 mg/kg[39]; **(f)** Plasma concentrations of cobimetinib after a single oral dose (3 or 10 mg/kg) and following 14 days of repeated dosing at 1, 3, and 10 mg/kg (QD, oral administration)[40].S, simulation; D, published PK data in mouse.

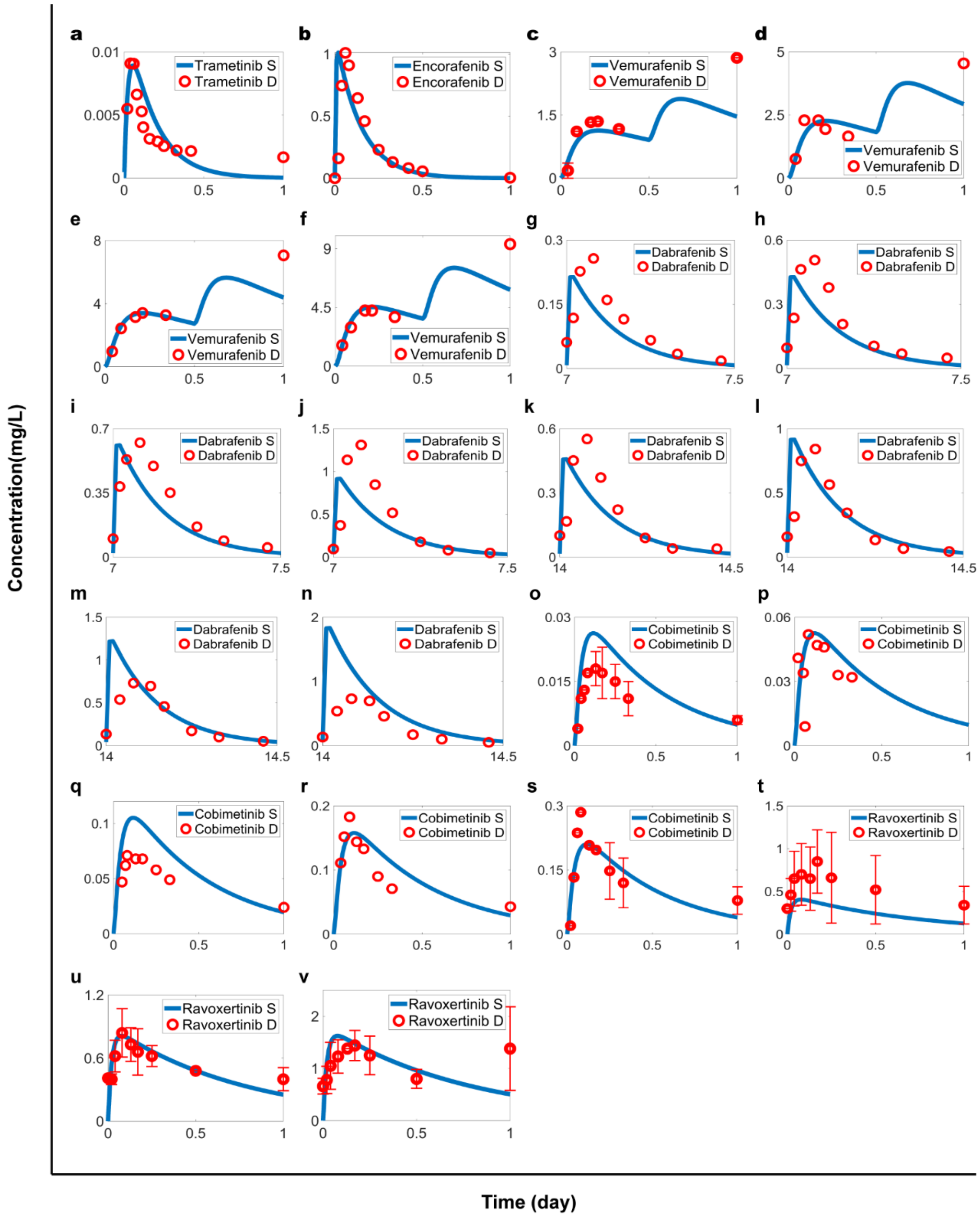

Supplementary Fig S7. QSP submodules describing PK of various drugs in human (Part 1), related to Figs. 6, 7 and 8. QSP submodules describing PK of various drugs in human In the QSP model, we incorporated PK submodules of various drugs in human that are calibrated against published data, including: **(a)** plasma concentrations of trametinib following a single oral dose of 2 mg[41]; **(b)** plasma concentrations of encorafenib following a single oral dose of 100 mg[42]; **(c-f)** plasma concentrations of vemurafenib following an oral dose of 240, 480, 720, or 960 mg, BID[43]; **(g-n)** plasma concentrations of dabrafenib following oral BID dosing at: 35, 70, 100, 150 mg (Day 8); and 75, 150, 200, 300 mg (Day 15) [44]; **(o-s)** plasma concentrations of cobimetinib following a single oral dose of 10,20,40,60, or 80 mg[45]; **(t-v)** plasma concentrations of ravoxertinib following a single oral dose of 50,100, or 200 mg[39]. S, simulation; D, published PK data in human.

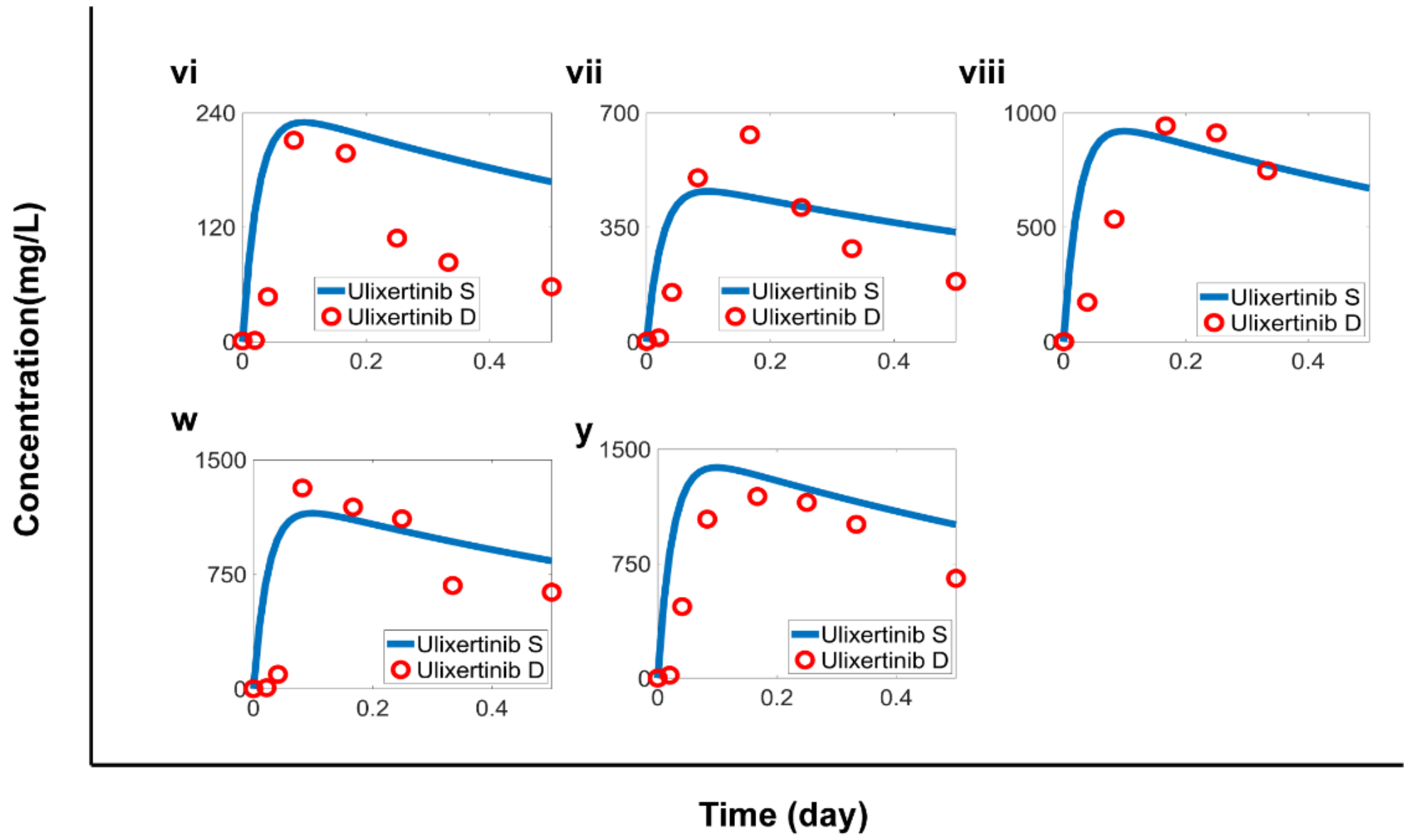

Supplementary Fig S8. QSP submodules describing PK of various drugs in human (Part 2), related to Figs. 6, 7 and 8. QSP submodules describing PK of various drugs in human In the QSP model, we incorporated PK submodules of various drugs in human that are calibrated against published data, including: **(vi-y)** plasma concentrations of ulixertinib following an oral dose of 150, 300, 600, 750, or 900 mg, BID[46]. S, simulation; D, published PK data in human.

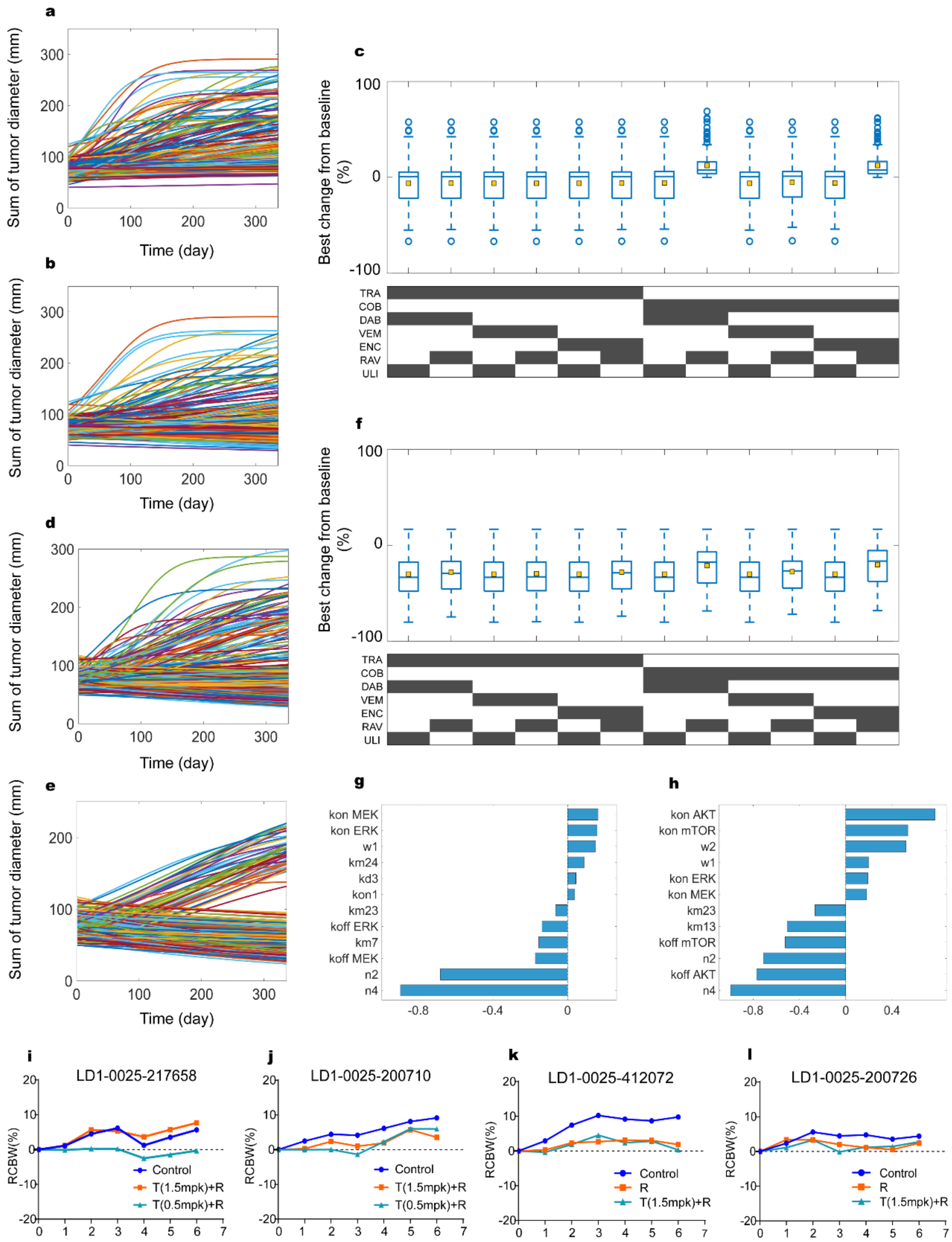
a
Sum of tumor diameter (mm)
Time (day)
b
Sum of tumor diameter (mm)
Time (day)
c
Best change from baseline (%)
TRA
COB
DAB
VEM
ENC
RAV
ULI
d
Sum of tumor diameter (mm)
Time (day)
f
Best change from baseline (%)
TRA
COB
DAB
VEM
ENC
RAV
ULI
e
Sum of tumor diameter (mm)
Time (day)
g
kon MEK
kon ERK
w1
km24
kd3
kon1
km23
koff ERK
km7
koff MEK
n2
n4
h
kon AKT
kon mTOR
w2
w1
kon ERK
kon MEK
km23
km13
koff mTOR
n2
koff AKT
n4
i
LD1-0025-217658
RCBW(%)
Control
T(1.5mpk)+R
T(0.5mpk)+R
j
LD1-0025-200710
RCBW(%)
Control
T(1.5mpk)+R
T(0.5mpk)+R
k
LD1-0025-412072
RCBW(%)
Control
R
T(1.5mpk)+R
l
LD1-0025-200726
RCBW(%)
Control
R
T(1.5mpk)+R

Supplementary Fig S9. From in silico predictions to in vivo validation: optimization of mono- and combination therapy regimens for Class II/III BRAF-mutant NSCLC through virtual clinical trials and MiniPDX experiments, related to Figs 7 and 8. Using the model-based virtual patient population, individual changes in tumor SLD was simulated for **(a)** vemurafenib (RAFi) monotherapy and **(b)** dabrafenib (RAFi) plus trametinib (MEKi) combination in Class II patients. **(c)** Simulated population-level efficacy of combinations involving RAFi, MEKi, and ERKi in Class II patients (displaying averaged SLD% change under each combination regimen). Individual changes in tumor SLD was also simulated for Class III patient: **(d)** vemurafenib (RAFi) monotherapy, **(e)** dabrafenib (RAFi) plus trametinib (MEKi) combination, and **(f)** different RAFi/MEKi/ERKi combinations. Key parameters driving the response to trametinib (MEKi) in **(g)** Class I and **(h)**Class III mutation identified by global sensitivity analysis using the partial rank correlation coefficient method. In the box plots (c and f), the yellow square represents the mean value. **(i-l)** In *vivo* MiniPDX validation of specific combination regimens in two **(i-j)** Class II and **(k-l)** Class III BRAF-mutant experimental models (RCBW indicates relative mean changes in body weight); n=6 for each MiniPDX model. T, trametinib. R, ravoxertinib.

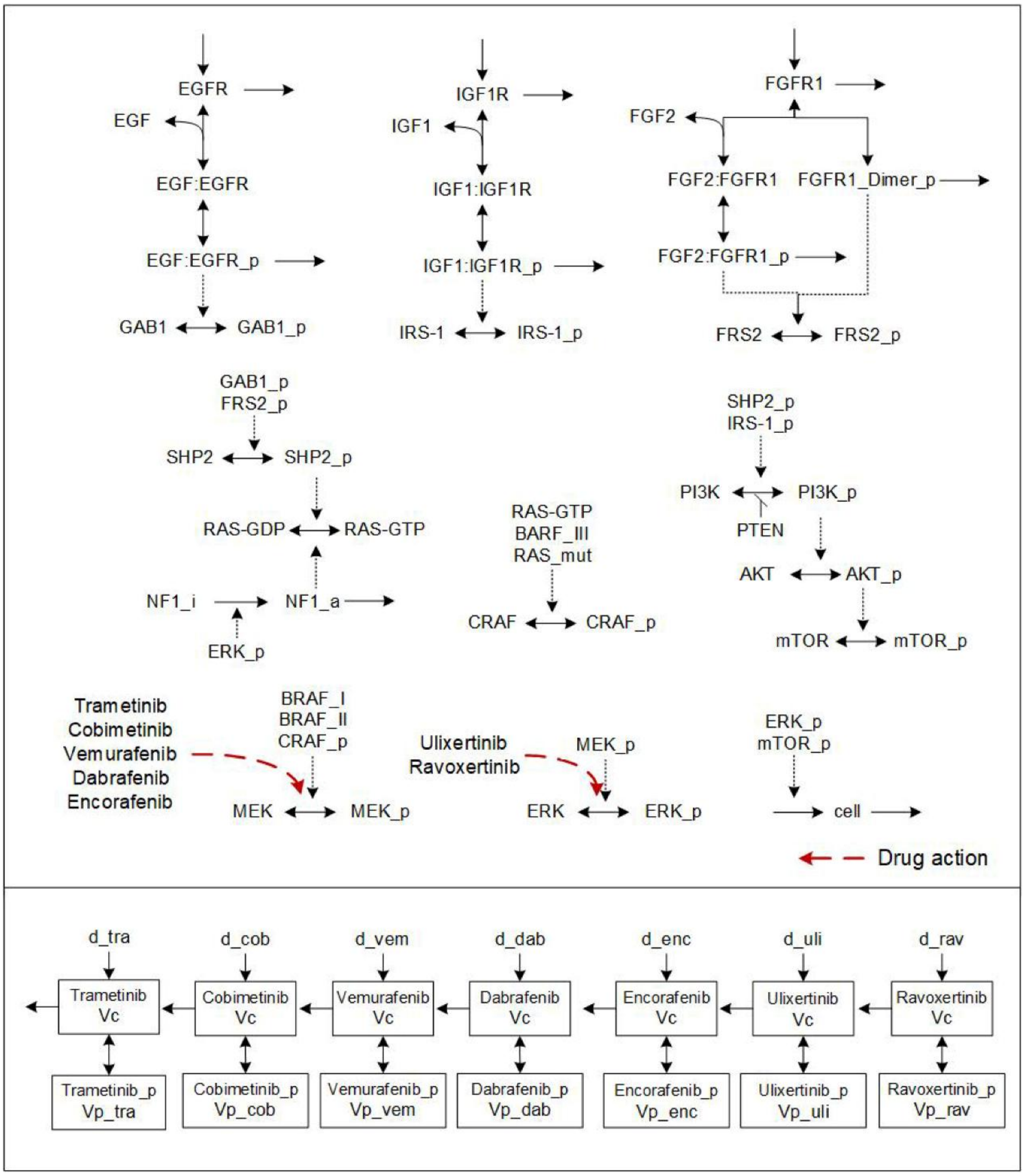

Supplementary Fig S10. Detailed diagram of the QSP model structure, related to Fig. 1. Unique model species and reaction fluxes were shown with labels. See more details in Supplemental Table S1.

Supplemental References

1 Zhou L, Wang X, Lu J, Fu X, Li Y. EGFR transcriptionally upregulates UTX via STAT3 in non-small cell lung cancer. J Cancer Res Clin Oncol 2021; 148: 309-19.

2 Wang T, Zhang J, Wang S, Sun X, Wang D, Gao Y, *et al*. The exon 19-deleted EGFR undergoes ubiquitylation-mediated endocytic degradation via dynamin activity-dependent and -independent mechanisms. Cell Commun Signal 2018; 16: 40.

3 Kiyatkin A, Aksamitiene E, Markevich NI, Borisov NM, Hoek JB, Kholodenko BN. Scaffolding protein Grb2-associated binder 1 sustains epidermal growth factor-induced mitogenic and survival signaling by multiple positive feedback loops. J Biol Chem 2006; 281: 19925-38.

4 Zhang L, Bewick M, Lafrenie RM. EGFR and ErbB2 differentially regulate Raf-1 translocation and activation. Lab Invest 2002; 82: 71-8.

5 Ahmed TA, Adamopoulos C, Karoulia Z, Wu X, Sachidanandam R, Aaronson SA, *et al*. SHP2 Drives Adaptive Resistance to ERK Signaling Inhibition in Molecularly Defined Subsets of ERK-Dependent Tumors. Cell Rep 2019; 26.

6 Huang M-H, Lee J-H, Chang Y-J, Tsai H-H, Lin Y-L, Lin AM-Y, *et al*. MEK inhibitors reverse resistance in epidermal growth factor receptor mutation lung cancer cells with acquired resistance to gefitinib. Mol Oncol 2012; 7: 112-20.

7 Nakashima M, Adachi S, Yasuda I, Yamauchi T, Kawaguchi J, Hanamatsu T, *et al*. Inhibition of Rho-associated coiled-coil containing protein kinase enhances the activation of epidermal growth factor receptor in pancreatic cancer cells. Mol Cancer 2011; 10: 79.

8 Borisov N, Aksamitiene E, Kiyatkin A, Legewie S, Berkhout J, Maiwald T, *et al*. Systems-level interactions between insulin-EGF networks amplify mitogenic signaling. Mol Syst Biol 2009; 5: 256.

9 Lin VC-H, Kuo P-T, Lin Y-C, Chen Y, Hseu Y-C, Yang H-L, *et al*. Penta-O-galloyl-β-D-glucose suppresses EGF-induced eIF3i expression through inhibition of the PI3K/AKT/mTOR pathway in prostate cancer cells. J Agric Food Chem 2014; 62: 8990-6.

10 Ma J, Dong C, Cao Y-Z, Ma B-L. Dual Target of EGFR and mTOR Suppresses Triple-Negative Breast Cancer Cell Growth by Regulating the Phosphorylation of mTOR Downstream Proteins. Breast Cancer (Dove Med Press) 2023; 15: 11-24.

11 Cao H, Dong W, Qu X, Shen H, Xu J, Zhu L, *et al*. Metformin Enhances the Therapy Effects of Anti-IGF-1R mAb Figitumumab to NSCLC. Sci Rep 2016; 6: 31072.

12 Shen H, Fang Y, Dong W, Mu X, Liu Q, Du J. IGF-1 receptor is down-regulated by sunitinib induces MDM2-dependent ubiquitination. FEBS Open Bio 2011; 2: 1-5.

13 Choucair A, Pham TH, Omarjee S, Jacquemetton J, Kassem L, Trédan O, *et al*. The arginine methyltransferase PRMT1 regulates IGF-1 signaling in breast cancer. Oncogene 2019; 38: 4015-27.
14 Moser C, Schachtschneider P, Lang SA, Gaumann A, Mori A, Zimmermann J, *et al*. Inhibition of insulin-like growth factor-I receptor (IGF-IR) using NVP-AEW541, a small molecule kinase inhibitor, reduces orthotopic pancreatic cancer growth and angiogenesis. Eur J Cancer 2008; 44: 1577-86.
15 Jia G, Mitra AK, Gangahar DM, Agrawal DK. Insulin-like growth factor-1 induces phosphorylation of PI3K-Akt/PKB to potentiate proliferation of smooth muscle cells in human saphenous vein. Exp Mol Pathol 2010; 89: 20-6.
16 Wang K, Ji W, Yu Y, Li Z, Niu X, Xia W, *et al*. FGFR1-ERK1/2-SOX2 axis promotes cell proliferation, epithelial-mesenchymal transition, and metastasis in FGFR1-amplified lung cancer. Oncogene 2018; 37: 5340-54.
17 Yuan H, Li Z-M, Shao J, Ji W-X, Xia W, Lu S. FGF2/FGFR1 regulates autophagy in FGFR1-amplified non-small cell lung cancer cells. J Exp Clin Cancer Res 2017; 36: 72.
18 Cirit M, Grant KG, Haugh JM. Systemic perturbation of the ERK signaling pathway by the proteasome inhibitor, MG132. PLoS One 2012; 7: e50975.
19 Lau M-T, So W-K, Leung PCK. Fibroblast growth factor 2 induces E-cadherin down-regulation via PI3K/Akt/mTOR and MAPK/ERK signaling in ovarian cancer cells. PLoS One 2013; 8: e59083.
20 Gerosa L, Chidley C, Froehlich F, Sanchez G, Lim SK, Muhlich J, *et al*. Sporadic ERK pulses drive non-genetic resistance in drug-adapted BRAFV600E melanoma cells. BioRxiv 2019: 762294.
21 Negrao MV, Raymond VM, Lanman RB, Robichaux JP, He J, Nilsson MB, *et al*. Molecular Landscape of BRAF-Mutant NSCLC Reveals an Association Between Clonality and Driver Mutations and Identifies Targetable Non-V600 Driver Mutations. J Thorac Oncol 2020; 15: 1611-23.
22 Rustgi N, Maria A, Toumbacaris N, Zhao H, Kargus K, Bryant M, *et al*. Combined RAF and MEK Inhibition to Treat Activated Non-V600 BRAF-Altered Advanced Cancers. Oncologist 2024; 29: 15-24.
23 Lin L, Asthana S, Chan E, Bandyopadhyay S, Martins MM, Olivas V, *et al*. Mapping the molecular determinants of BRAF oncogene dependence in human lung cancer. Proc Natl Acad Sci U S A 2014; 111: E748-E57.
24 Kotani H, Adachi Y, Kitai H, Tomida S, Bando H, Faber AC, *et al*. Distinct dependencies on receptor tyrosine kinases in the regulation of MAPK signaling between BRAF V600E and non-V600E mutant lung cancers. Oncogene 2018; 37: 1775-87.
25 Cotto-Rios XM, Agianian B, Gitego N, Zacharioudakis E, Giricz O, Wu Y, *et al*. Inhibitors of BRAF dimers using an allosteric site. Nat Commun 2020; 11: 4370.
26 Noeparast A, Giron P, De Brakeleer S, Eggermont C, De Ridder U, Teugels E, *et al*. Type II RAF inhibitor causes superior ERK pathway suppression

compared to type I RAF inhibitor in cells expressing different BRAF mutant types recurrently found in lung cancer. Oncotarget 2018; 9: 16110-23.

27 Yao Z, Yaeger R, Rodrik-Outmezguine VS, Tao A, Torres NM, Chang MT, *et al*. Tumours with class 3 BRAF mutants are sensitive to the inhibition of activated RAS. Nature 2017; 548: 234-8.

28 Rajkumar S, Berry D, Heney KA, Strong C, Ramsay L, Lajoie M, *et al*. Melanomas with concurrent BRAF non-p.V600 and NF1 loss-of-function mutations are targetable by BRAF/MEK inhibitor combination therapy. Cell Rep 2022; 39: 110634.

29 Chen Y, Sang Y, Li S, Xue J, Chen M, Hong S, *et al*. The ERK inhibitor GDC-0994 selectively inhibits growth of BRAF mutant cancer cells. Transl Oncol 2024; 45: 101991.

30 Cui H, Wang Q, Miller DD, Li W. The Tubulin Inhibitor VERU-111 in Combination With Vemurafenib Provides an Effective Treatment of Vemurafenib-Resistant A375 Melanoma. Front Pharmacol 2021; 12: 637098.

31 King AJ, Arnone MR, Bleam MR, Moss KG, Yang J, Fedorowicz KE, *et al*. Dabrafenib; preclinical characterization, increased efficacy when combined with trametinib, while BRAF/MEK tool combination reduced skin lesions. PLoS One 2013; 8: e67583.

32 Rheault TR, Stellwagen JC, Adjabeng GM, Hornberger KR, Petrov KG, Waterson AG, *et al*. Discovery of Dabrafenib: A Selective Inhibitor of Raf Kinases with Antitumor Activity against B-Raf-Driven Tumors. ACS Med Chem Lett 2013; 4: 358-62.

33 Biotech L. LIDE PMed-TRIAL PDX Model Database, <https://pmed.lidebiotech.com:8000>.

34 Dankner M, Lajoie M, Moldoveanu D, Nguyen T-T, Savage P, Rajkumar S, *et al*. Dual MAPK Inhibition Is an Effective Therapeutic Strategy for a Subset of Class II BRAF Mutant Melanomas. Clin Cancer Res 2018; 24: 6483-94.

35 Wei B-R, Hoover SB, Peer CJ, Dwyer JE, Adissu HA, Shankarappa P, *et al*. Efficacy, Tolerability, and Pharmacokinetics of Combined Targeted MEK and Dual mTORC1/2 Inhibition in a Preclinical Model of Mucosal Melanoma. Mol Cancer Ther 2020; 19: 2308-18.

36 Karbasi AB, Barfuss JD, Morgan TC, Collins D, Costenbader DA, Dennis DG, *et al*. Sol-moiety: Discovery of a water-soluble prodrug technology for enhanced oral bioavailability of insoluble therapeutics. Nat Commun 2024; 15: 8487.

37 Henderson CJ, Kapelyukh Y, Scheer N, Rode A, McLaren AW, MacLeod AK, *et al*. An Extensively Humanized Mouse Model to Predict Pathways of Drug Disposition and Drug/Drug Interactions, and to Facilitate Design of Clinical Trials. Drug Metab Dispos 2019; 47: 601-15.

38 Suresh PS, Jairam RK, Chandrasekhar DV, Vinod AB, Hiremath RA, Raj A, *et al*. Prediction of Human Pharmacokinetics of Ulixertinib, a Novel ERK1/2 Inhibitor from Mice, Rats, and Dogs Pharmacokinetics. Eur J Drug Metab Pharmacokinet 2018; 43: 453-60.

39 Blake JF, Burkard M, Chan J, Chen H, Chou K-J, Diaz D, *et al*. Discovery of (S)-1-(1-(4-Chloro-3-fluorophenyl)-2-hydroxyethyl)-4-(2-((1-methyl-1H-pyrazol-5-yl)amino)pyrimidin-4-yl)pyridin-2(1H)-one (GDC-0994), an Extracellular Signal-Regulated Kinase 1/2 (ERK1/2) Inhibitor in Early Clinical Development. J Med Chem 2016; 59: 5650-60.

40 Wong H, Vernillet L, Peterson A, Ware JA, Lee L, Martini J-F, *et al*. Bridging the gap between preclinical and clinical studies using pharmacokinetic-pharmacodynamic modeling: an analysis of GDC-0973, a MEK inhibitor. Clin Cancer Res 2012; 18: 3090-9.

41 Leonowens C, Pendry C, Bauman J, Young GC, Ho M, Henriquez F, *et al*. Concomitant oral and intravenous pharmacokinetics of trametinib, a MEK inhibitor, in subjects with solid tumours. Br J Clin Pharmacol 2014; 78: 524-32.

42 Piscitelli J, Hens B, Tomaszewska I, Wollenberg L, Litwiler K, McAllister M, *et al*. Effect of Food and a Proton-Pump Inhibitor on the Absorption of Encorafenib: An In Vivo-In Vitro-In Silico Approach. Mol Pharm 2023; 20: 2589-99.

43 Grippo JF, Zhang W, Heinzmann D, Yang KH, Wong J, Joe AK, *et al*. A phase I, randomized, open-label study of the multiple-dose pharmacokinetics of vemurafenib in patients with BRAF V600E mutation-positive metastatic melanoma. Cancer Chemother Pharmacol 2013; 73: 103-11.

44 Falchook GS, Long GV, Kurzrock R, Kim KB, Arkenau HT, Brown MP, *et al*. Dose selection, pharmacokinetics, and pharmacodynamics of BRAF inhibitor dabrafenib (GSK2118436). Clin Cancer Res 2014; 20: 4449-58.

45 Rosen LS, LoRusso P, Ma WW, Goldman JW, Weise A, Colevas AD, *et al*. A first-in-human phase I study to evaluate the MEK1/2 inhibitor, cobimetinib, administered daily in patients with advanced solid tumors. Invest New Drugs 2016; 34: 604-13.

46 Sullivan RJ, Infante JR, Janku F, Wong DJL, Sosman JA, Keedy V, *et al*. First-in-Class ERK1/2 Inhibitor Ulixertinib (BVD-523) in Patients with MAPK Mutant Advanced Solid Tumors: Results of a Phase I Dose-Escalation and Expansion Study. Cancer Discov 2017; 8: 184-95.
